# Precise editing of *CLAVATA* genes in *Brassica napus* L. regulates multilocular silique development

**DOI:** 10.1111/pbi.12872

**Published:** 2018-01-19

**Authors:** Yang Yang, Kaiyu Zhu, Huailin Li, Shaoqing Han, Qingwei Meng, Shahid Ullah Khan, Chuchuan Fan, Kabin Xie, Yongming Zhou

**Affiliations:** ^1^ National Key Laboratory of Crop Genetic Improvement Huazhong Agricultural University Wuhan China

**Keywords:** *Brassica napus*, genome editing, CRISPR/Cas9, *CLAVATA* genes, multilocular silique

## Abstract

Multilocular silique is a desirable agricultural trait with great potential for the development of high‐yield varieties of *Brassica*. To date, no spontaneous or induced multilocular mutants have been reported in *Brassica napus*, which likely reflects its allotetraploid nature and the extremely low probability of the simultaneous random mutagenesis of multiple gene copies with functional redundancy. Here, we present evidence for the efficient knockout of rapeseed homologues of *CLAVATA3* (*CLV3*) for a secreted peptide and its related receptors *CLV1* and *CLV2* in the *CLV* signalling pathway using the CRISPR/Cas9 system and achieved stable transmission of the mutations across three generations. Each *BnCLV* gene has two copies located in two subgenomes. The multilocular phenotype can be recovered only in knockout mutations of both copies of each *BnCLV* gene, illustrating that the simultaneous alteration of multiple gene copies by CRISPR/Cas9 mutagenesis has great potential in generating agronomically important mutations in rapeseed. The mutagenesis efficiency varied widely from 0% to 48.65% in T_0_ with different single‐guide RNAs (sgRNAs), indicating that the appropriate selection of the sgRNA is important for effectively generating indels in rapeseed. The double mutation of *BnCLV3* produced more leaves and multilocular siliques with a significantly higher number of seeds per silique and a higher seed weight than the wild‐type and single mutant plants, potentially contributing to increased seed production. We also assessed the efficiency of the horizontal transfer of Cas9/gRNA cassettes by pollination. Our findings reveal the potential for plant breeding strategies to improve yield traits in currently cultivated rapeseed varieties.

## Introduction

Rapeseed (*Brassica napus* L., AACC, 2*n* = 38), one of the most important oil crops worldwide, provides edible oil for human diets, protein‐rich feed for animals and raw materials for industrial processes, such as biodiesel production. Achieving high yields and genetic improvements has always been the major goal in rapeseed production. The siliques of oilseed rape contain seeds that serve not only as the productive organs for the life cycle but also as storage compartments for oils and proteins, which are the predominant products of this crop. Therefore, the silique and its related traits, that is, the number of siliques per plant, the number of seeds per silique (NSS) and the seed weight (SW), are important factors for improving yield (Liu, 2000).

Similar to *Arabidopsis,* the silique of *B. napus* develops from the gynoecium, which typically comprises two carpels that are separated by a false septum, and thus has two locules (bilocular). A few multilocular (more than two carpels) lines of *Brassica* have been identified in nature, such as the multilocular *yellow sarson* in *B. rapa* and Santong, Silun, Duoshi in *B. juncea* (Liu, 2000). A multilocular silique is a desirable agricultural trait that has great potential in developing high‐yield varieties of *Brassica* due to the potentially greater NSS and better shatter resistance to avoid seed loss during mechanical harvest (Katiyar *et al*., 1998; Lv *et al*., 2012; Varshney, 1987; Xiao *et al*., 2013; Xu *et al*., 2013). However, only a few studies have investigated the multilocular trait in *B. napus* due to the lack of mutants with stable multilocular traits. Thus far, no multilocular trait has been applied to rapeseed breeding.

One and two recessive nuclear genes are responsible for the multilocular trait in *B. rapa* and *B. juncea*, respectively (He *et al*., 2003; Lv *et al*., 2012; Xiao *et al*., 2013). A single‐nucleotide mutation in *CLAVATA3* (*CLV3*) gene homologue and insertion of a copia‐LTR retrotransposable element in *CLAVATA1* (*CLV1*) gene homologue interrupt the function of the target genes and control the multilocular trait in *B. rapa* and *B. juncea*, respectively (Fan *et al*., 2014; Xu *et al*., 2017). In *Arabidopsis*,* CLV3* acts as a small secreted peptide that interacts with a CLV1‐CLV2‐CORYNE (CRN)‐RECEPTOR‐LIKE PROTEIN KINASE2 (RPK2) receptor kinase‐mediated pathway to repress the expression of the stem cell‐promoting homeodomain transcription factor *WUSCHEL* in shoot apical meristems (SAMs) (Clark *et al*., 1993, 1997; Jeong *et al*., 1999; Kayes and Clark, 1998; Kinoshita *et al*., 2010; Müller *et al*., 2008). CLV1 encodes a member of the leucine‐rich repeat (LRR) receptor kinase family (Clark *et al*., 1997); CLV2 encodes a LRR receptor‐like protein lacking a cytoplasmic domain and acts together with a membrane‐associated protein kinase, CRN/SUPPRESSOR OF LLP1 2 (SOL2), to transmit the CLV3 signal (Jeong *et al*., 1999). RPK2 is another key receptor‐like kinase in the *CLV* pathway (Kinoshita *et al*., 2010). Mutations in *CLV* pathway genes result in expanded SAMs, an increased number of floral organs and multilocular siliques (Clark *et al*., 1995; Fletcher *et al*., 1999). The *CLV* pathway is functionally conserved in plants. In tomato, mutations in the homologues of *CLV1*,* CLV2* and *CLV3* increase the locule number and thus increase the fruit size (Xu *et al*., 2015). Mutations in the homologous genes in the *CLV* signalling pathway in maize and rice, such as *FASCIATED EAR2* (*FAE2*), *THICK TASSEL DWARF1* (*TD1*), *FLORAL ORGAN NUMBER1* (*FON1*) and *FLORAL ORGAN NUMBER4* (*FON4*), also increase the kernel row number in maize and the seed number per inflorescence in rice (Bommert *et al*., 2005, 2013; Chu *et al*., 2006; Suzaki *et al*., 2004; Taguchi‐Shiobara *et al*., 2001). Thus, *CLV* pathway genes are attractive targets for the genomic engineering of *Brassica* species to improve yield‐related traits.

*Brassica napus* resulted from a recent allopolyploidy between ancestors of *B. rapa* (2*n* = 20, AA) and *B. oleracea* (2*n* = 18, CC). In addition to more ancient polyploidization events, this recent allopolyploidy conferred an aggregate 72× genome multiplication since the origin of angiosperms (Chalhoub *et al*., 2014). Therefore, obtaining rapeseed mutants is challenging due to the high genetic redundancy, and genetic analyses are critical for determining gene function in both basic and applied research studies. Therefore, technologies that can target one specific copy or several homologous gene copies are needed to characterize and improve the agronomic traits of rapeseed.

Recently, various genome‐editing methods, particularly methods involving sequence‐specific nucleases (SSNs) for creating targeted double‐strand breaks (DSBs), have emerged as major breakthroughs in site‐specific genome editing (Cong *et al*., 2013; Li *et al*., 2011; Wood *et al*., 2011). In particular, CRISPR/CRISPR‐associated 9 (CRISPR/Cas9) is considered the most simple and effective SSN developed thus far and has been used for genome editing in major crops, including rice (Jiang *et al*., 2013; Miao *et al*., 2013; Shan *et al*., 2013), sorghum (Jiang *et al*., 2013), tobacco (Gao *et al*., 2015), wheat (Shan *et al*., 2013; Wang *et al*., 2014), maize (Liang *et al*., 2014), barley (Lawrenson *et al*., 2015), cotton (Wang *et al*., 2017), tomato (Brooks *et al*., 2014), soya bean (Li *et al*., 2015) and camelina (Jiang *et al*., 2017). To date, few studies have reported successful site‐directed mutagenesis using the Cas9/single‐guide RNA (sgRNA) system in rapeseed (Braatz *et al*., 2017). Therefore, genome‐editing methods in rapeseed have not been fully established, and the further relevant studies are required for the utilization of these methods on a practical level.

Here, we used the CRISPR/Cas9 system to generate efficient knockouts of three key genes in the *CLV* signalling pathway with stable transformation in rapeseed. The CRISPR/Cas9‐induced mutations of both copies of each *BnCLV* gene could result in multilocular siliques. In particular, the double mutation of *BnCLV3* produced heritable multilocular siliques that could increase seed production. Thus, the CRISPR/Cas9 system can advance rapeseed functional genomic research studies and has the potential to improve plant breeding strategies to yield beneficial traits in currently cultivated varieties.

## Results

### Design of sgRNAs to knock out homologues of the *CLV* genes in *B. napus*

We have shown that *B. rapa* harbouring loss‐of‐function *clv3* alleles produces more locules and higher seed yield (Fan *et al*., 2014). Thus, modifications of *CLV3* in *B. napus* may provide an opportunity to breed high‐yield varieties. However, attempts to introduce *clv3* null alleles from *B. rapa* into *B. napus* do not result in the desired traits due to the multiple dominant *CLV3* alleles in the subgenomes. According to the released rapeseed genome information, *B. napus* cultivar Darmor‐bzh contains three *CLV3* copies, that is, *BnA04.CLV3* (*BnaA04g15710D*), *BnC04.CLV3* (*BnaC04g38990D*) and *BnC02.CLV3* (*BnaC02g15230D*) (http://www.genoscope.cns.fr/brassicanapus/). We confirmed the sequences of *BnA04.CLV3* and *BnC04.CLV3* in the *B. napus* pure line J9707, which is amenable to *Agrobacterium*‐mediated transformation; however, we were unable to amplify *BnC02.CLV3*. The result of genomic Southern blotting analysis verified only two copies of *BnCLV3* gene in J9707 and the Darmor‐bzh reference genome (Figure S1). Further analysis of the recently released genome of *B. napus* cultivar ZS11 also showed that it contains only the copies of *BnA04.CLV3* and *BnC04.CLV3* (Sun *et al*., 2017). Collectively, these results indicated the lack of *BnC02.CLV3* in the *B*. *napus* genome. *BnA04.CLV3* and *BnC04.CLV3* are 94.8% and 97.8% identical at the nucleotide and protein levels, respectively, suggesting that these genes may share similar functions. After careful analysis on the sequences of these two *BnCLV3* copies, we were able to distinguish the origins of these copies by means of several single‐nucleotide polymorphisms (SNPs) (Figure S2).

**Figure 1:**
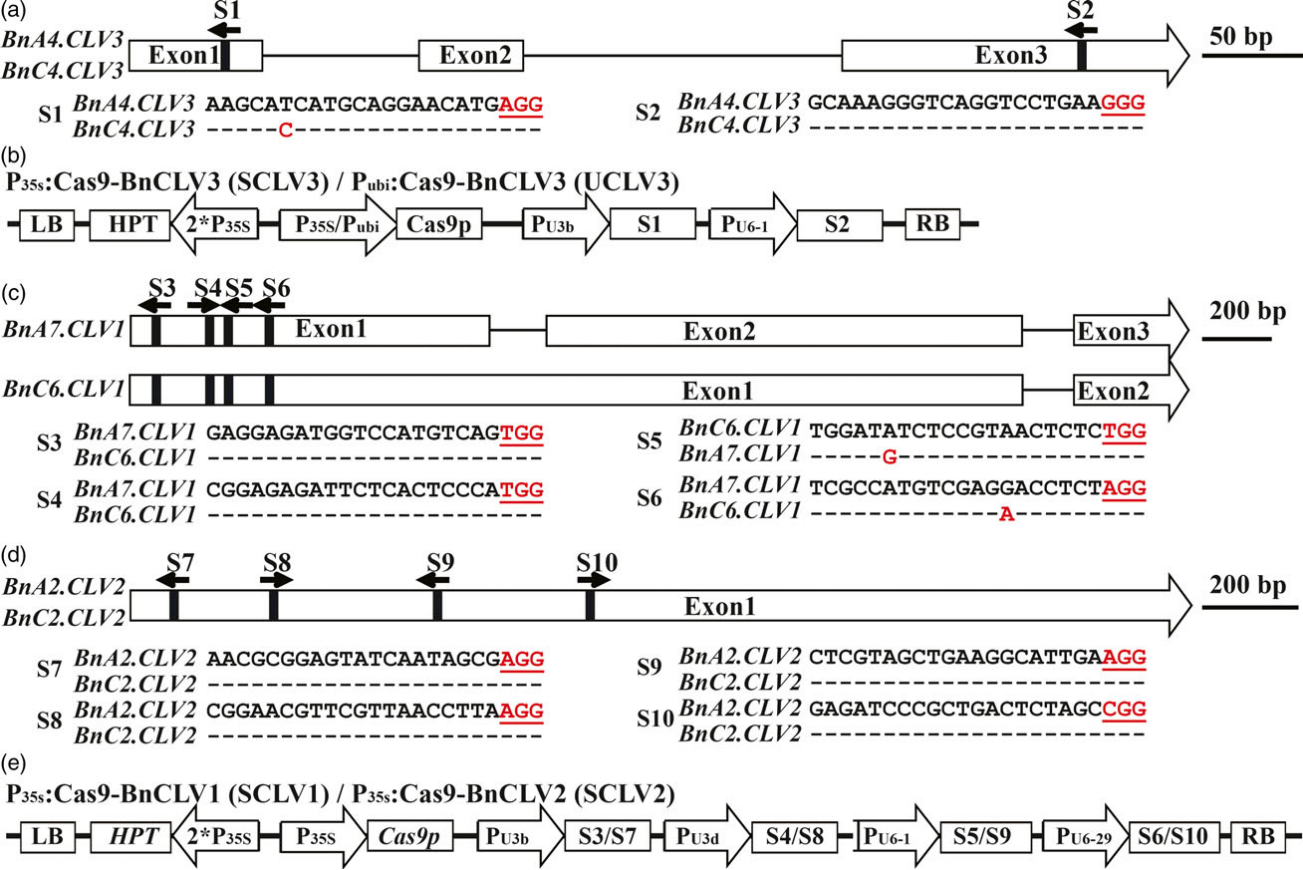
*BnCLV* gene models with target sequences and schematics of binary plasmid vectors. (a) The *BnCLV3* gene model includes three exons (white box) separated by two introns (represented by the solid line). The vertical line in the gene model indicates the target site, and the arrow indicates the sgRNA direction. The target sequences are shown with the PAM highlighted in red. (b) The constructs of SCLV3 and UCLV3 house the following: a hygromycin resistance cassette consisting of the hygromycin phosphotransferase coding sequence driven by the cauliflower mosaic virus 35S promoter; a Cas9 expression cassette comprising the sequence encoding Cas9 driven by P_35S_ or a ubiquitin promoter from maize; and two sgRNAs S1 and S2 driven by the U3b and U6‐1 promoters from *Arabidopsis*, respectively. (c, b) The *BnCLV1* gene model with target sites S3 to S6 and the *BnCLV2* gene model with target sites S7 to S10. (e) The binary constructs SCLV1 and SCLV2 with four sgRNAs driven by the U3b, U3d, U6‐1 and U6‐29 promoters from *Arabidopsis*. [Colour figure can be viewed at http://wileyonlinelibrary.com]

To generate Cas9‐induced mutations in both copies of *BnCLV3*, two sgRNAs, that is, sgRNA1 (S1) and sgRNA2 (S2), that target the first exon and the C‐terminal conserved *CLV3*/*ESR*‐related (*CLE*) domain, respectively, were designed using the CRISPR‐P program (Lei *et al*., 2014; Figure 1a,b). The sgRNAs precisely matched all *CLV3* copies, except for *BnC04.CLV3*, which has a SNP located 15‐bp upstream of the corresponding protospacer adjacent motif (PAM), whereas sgRNA2 matched well with both copies of *BnCLV3* (Figure 1a). These two sgRNAs were expressed with P_U3b_ and P_U6‐1_. To determine which promoter was better for Cas9 protein expression in rapeseed, two binary constructs carrying Cas9p driven by different promoters, that is, P_ubi_:Cas9‐BnCLV3 (referred to as UCLV3) and P_35s_:Cas9‐BnCLV3 (referred to as SCLV3), were generated based on the CRISPR/Cas9 multiplex genome‐editing vector as previously described by Ma *et al*. (2015b).

The rapeseed homologues of *CLV1* and *CLV2* were also targeted for knockout in J9707. According to the rapeseed genome information, there are two copies for each gene, that is *BnA07.CLV1* (*BnaA07g32120D*) and *BnC06.CLV1* (*BnaC06g36500D*) for *BnCLV1* with 96.1% identity at the nucleotide level and *BnA02.CLV2* (*BnaA02g12070D*) and *BnC02.CLV2* (*BnaC02g45200D*) for *BnCLV2* with 95.3% identity at the nucleotide level. According to the sequence alignment of these two copies of the *BnCLV1* and *BnCLV2* genes, polymorphisms distinguished the origins of these gene copies (Figures S3 and S4).

Two binary constructs carrying four sgRNAs within each target gene with Cas9p driven by P_35S_, that is P_35s_:Cas9‐BnCLV1 (containing sgRNA3‐sgRNA6, referred to as SCLV1) and P_35s_:Cas9‐BnCLV2 (containing sgRNA7‐sgRNA10, referred to as SCLV2), were generated as previously described by Ma *et al*. (2015b). These sgRNAs matched well with both copies of *BnCLV1* and *BnCLV2*, except for S5 and S6, which contain an SNP located 15 bp upstream of the corresponding PAM motif in *BnA07.CLV1* and 7 bp upstream of the corresponding PAM motif in *BnC06.CLV1*, respectively (Figure 1c‐e). Each site was located in the 5′ portion of each gene and was deliberately selected to ensure that gene disruptions altering the reading frame would produce a translation product lacking enzymatic activity.

### Rapid identification of the edited lines using native PAGE screening

**Figure 2:**
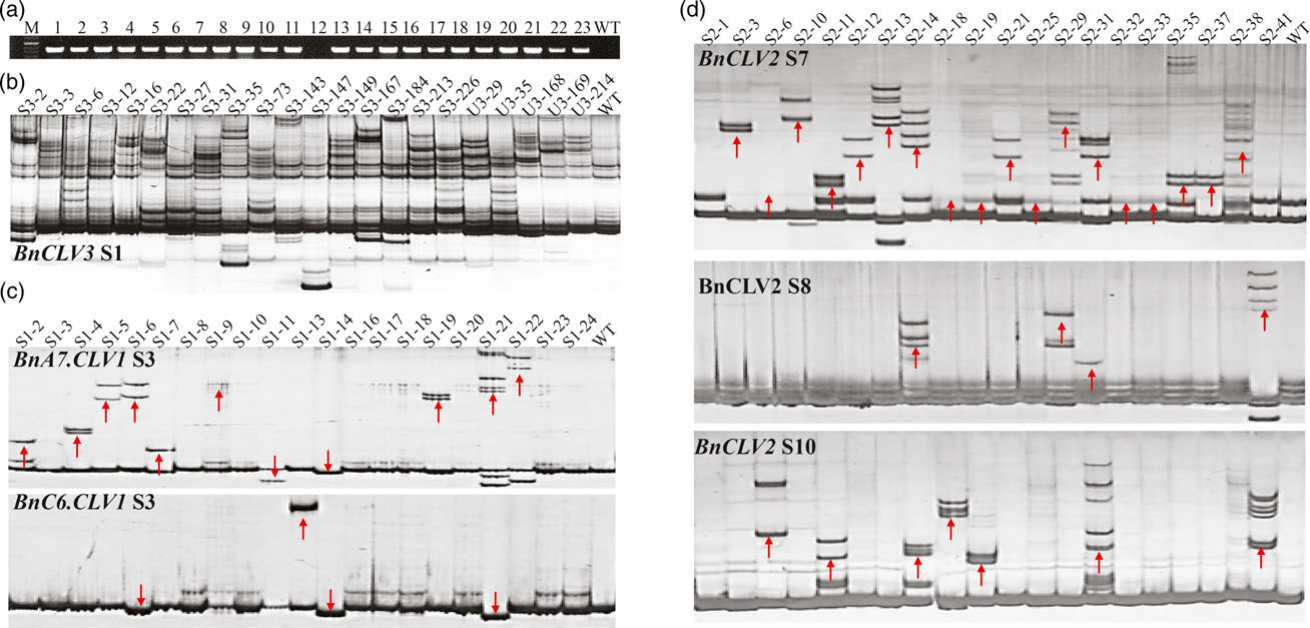
Detection of mutations in *BnCLVs* using PAGE method. (a) The transgenic positive detection in T_0_ regenerated plants via a PCR assay using *NPT II* gene‐specific primers and J9707 (WT) as a negative control. (b) Detection of the targeted mutations in T_0_ plants with the WT as a negative control. (c) Detection of mutations in different targets in the two *BnCLV1* copies in T_0_ plants with the WT as a negative control. (d) Detection of mutations in different targets in the two *BnCLV2* copies in T_0_ plants with the WT as a negative control. The numbers ‘S#‐#’ and ‘U#‐#’ above the PAGE gels represents the corresponding individual IDs ‘SCLV#‐#’ and ‘UCLV#‐#’, respectively. ‘S#’ represents the specific targets of *BnCLVs*. A red arrow indicates that the tested target has been edited. [Colour figure can be viewed at http://wileyonlinelibrary.com]

These four constructs were independently transformed into J9707 using *Agrobacterium*‐mediated transformation and generated 335, 366, 119 and 40 independent lines for SCLV3, UCLV3, SCLV1 and SCLV2, respectively. According to a PCR examination using *NPTII* gene‐specific primers, 74.6% (250/335), 66.7% (244/366), 84.9% (101/119) and 92.5% (37/40) of these T_0_ lines carried T‐DNA insertions (Figure 2a).

We performed polyacrylamide gel electrophoresis (PAGE) to identify the edited lines. Thus, the PCR products of each target site were denatured, renatured and subsequently separated using native PAGE. This method is based on the slower migration of heteroduplex DNA (with mutation) than homoduplex DNA (without mutation) on native PAGE (Zhu *et al*., 2014). Many plants displayed band profiles that differed from the profiles displayed by the wild‐type (WT) plants on the nondenaturing PAGE gels, indicating the presence of mutations in the target sites (Figure 2b‐d). According to the PAGE screening, 36/250 (14.4%), 15/244 (6.1%), 47/101 (46.5%) and 21/37 (56.8%) mutant plants were detected for SCLV3, UCLV3, SCLV1 and SCLV2, respectively (Tables S2‐S4). The editing efficiency of SCLV3 was higher than for UCLV3, suggesting that P_35s_ has higher activity than P_ubi_ in rapeseed. Interestingly, the contributions of the ten selected sgRNAs in directing Cas9 and mutating the target genes were not equal; the highest efficiency of mutagenesis was observed at S7 (48.7%) and S3 (46.5%), while an intermediate efficiency of mutagenesis was observed at S10 (24.3%) and S8 (13.5%), a lower efficiency of mutagenesis was observed at S1 (14.4% in SCLV3 and 6.2% in UCLV3) and S4&S5 (5.0%), and no mutation was detected at S2, S6 and S9 (Tables 1; S5 and S6), indicating that the appropriate selection of sgRNA pairs is important for effectively generating indels. The average mutagenesis efficiency of the sgRNAs driven by different promoters ranged widely from 0% to 47.6% (Table 1), indicating that not all promoters were effective in driving genome editing in rapeseed.

**Table 1:** Analysis of putative factors affecting editing efficiency at different sgRNA targets

Promoter	sgRNA	Target gene	Target sequence^a^	GC% content (without PAM)	Continuous matching between target and sgRNA sequence^b^	Editing efficiency^c^	Average efficiency
AtU3b	S1	*BnCLV3*	AAGCATCATGCAGGAACATGAGG	45.0%	3	14.4%	11.0%
	S4	*BnCLV1*	CGGAGAGATTCTCACTCCCATGG	55.0%	0	5.0%	
	S8	*BnCLV2*	CGGAACGTTCGTTAACCTTAAGG	45.0%	5	13.5%	
AtU3d	S3	*BnCLV1*	GAGGAGATGGTCCATGTCAGTGG	55.0%	8	46.5%	47.6%
	S7	*BnCLV2*	AACGCGGAGTATCAATAGCGAGG	50.0%	5	48.7%	
AtU6_1	S2	*BnCLV3*	GCAAAGGGTCAGGTCCTGAAGGG	55.0%	3	0.0%	0.0%
	S5	*BnCLV1*	TGGATATCTCCGTAACTCTCTGG	45.0%	7	0.0%	
	S9	*BnCLV2*	CTCGTAGCTGAAGGCATTGAAGG	50.0%	5	0.0%	
AtU6_29	S6	*BnCLV1*	TCGCCATGTCGAGGACCTCTAGG	60.0%	8	0.0%	12.2%
	S10	*BnCLV2*	GAGATCCCGCTGACTCTAGCCGG	60.0%	5	24.3%	

a The PAM is underlined.

b Counting the maximum number of continuous matching bases between target and sgRNA sequence.

c The percentage of edited plants over the total number of tested plants for the corresponding targets.

To determine whether PAGE‐based assays can readily be used to distinguish indels of any length, we generated a set of indel plasmids with deletions ranging from 1 to 8 bp. These indels were PCR‐amplified and evaluated using PAGE analysis. All the PCR amplicons with different deletion sizes, except for 1‐bp indels, could be distinguished from WT (Figure S5).

### Variety and frequency of mutations in *BnCLV3*

**Figure 3:**
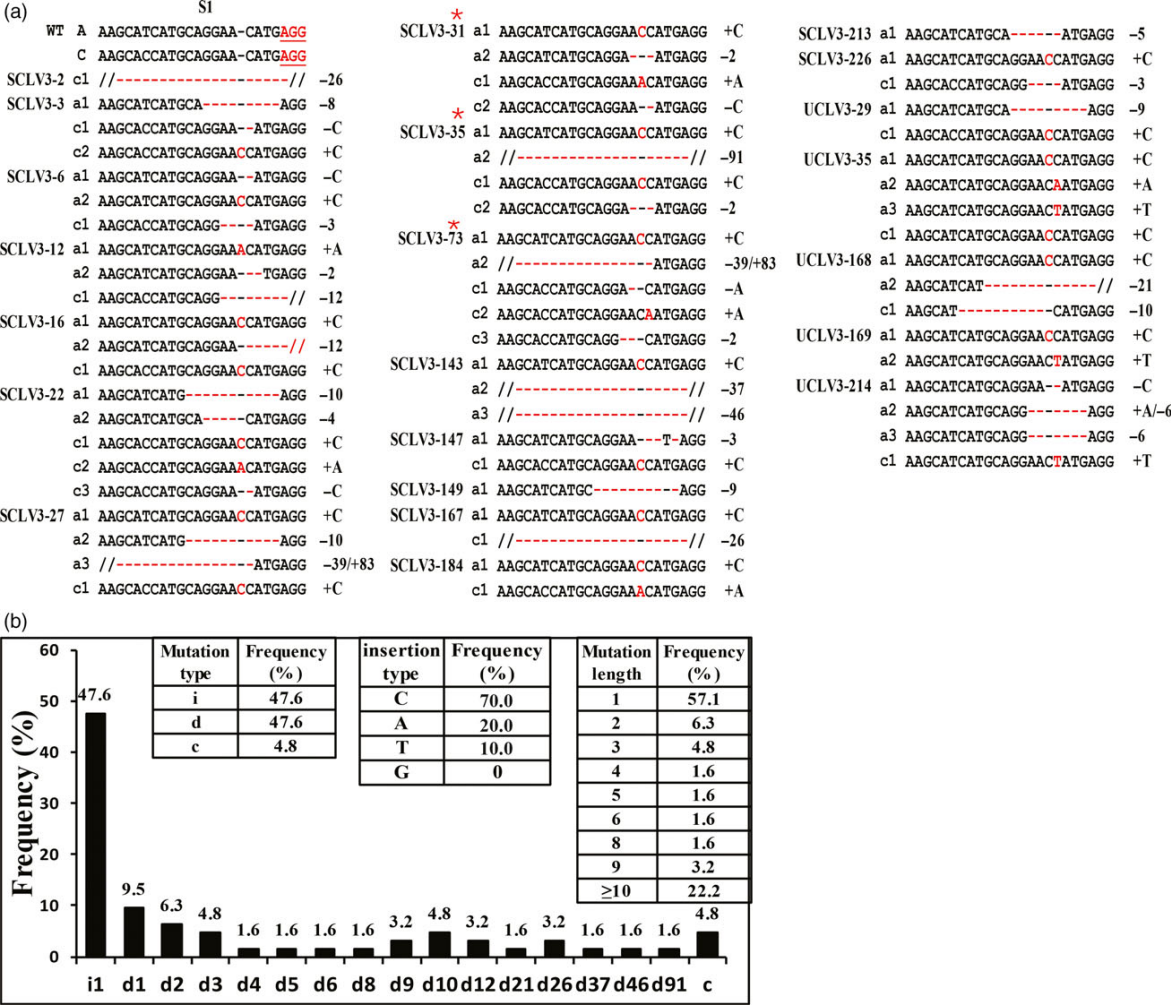
Detailed genotype analysis of mutations in *BnCLV3* on S1 in T_0_ generation. (a) Sequencing results of 22 editing T_0_ plants at the S1 site. Insertions and deletions are indicated in red font and with red hyphens, respectively. Edited plants with red stars have multilocular phenotypes. On the left, A and C and the WT allele of the *BnA04*.*CLV3* and *Bn0C4*.*CLV3* copies, respectively; a# and c# show the mutant allele numbers. ‘−’ and ‘+’ indicate the deletion and insertion of the indicated number of nucleotides, respectively; ‘−/+’ indicates the simultaneous deletion and insertion of the indicated number of nucleotides. (b) Mutation types and frequency at the S1 target site in 22 T_0_ plants. In the left insert table, the occurrence of deletions (d), insertions (i) and combined (c) mutation types is shown. In the right insert table, the frequency of different mutation lengths is shown. In the middle insert table, frequency of different insertion types is shown. *X*‐axis: d#, # of base pair (bp) deleted from the target site; i#, # of bp inserted at the target site; c#, combined mutation. [Colour figure can be viewed at http://wileyonlinelibrary.com]

To confirm the PAGE screening results, the PCR products from 22 edited lines of *BnCLV3* were sequenced. Various mutations, including the insertion and deletion of different nucleotides, were produced at the S1 target in all lines (Figure 3a), indicating that PAGE‐based screening is an efficient and simple method for identifying edited lines. Interestingly, S1 simultaneously targeted both copies of *BnCLV3,* even though the copy paired with the *BnC04.CLV3* target had one mismatch. This finding is consistent with the reported Cas9 specificity in targeting DNA sites with 1–5 mismatches proximal to the PAM. Of the 22 T_0_ lines, 17 lines were double mutants of *BnCLV3*, while the mutations in four lines and one line were restricted to *BnA04.CLV3* and *BnC04.CLV3*, respectively (Table S7). Of all the mutations examined, nearly half (48.7%, 19/39) of the *BnCLV3* loci were putatively heterozygous mutations, 25.6% (10/39) of the loci were chimeric mutations, 20.5% (8/39) of the loci were putatively bi‐allelic mutations, and 5.1% (2/39) of the loci were putatively homozygous mutations (Table S7). According to the allele mutation types, 47.6% of the mutations were nucleotide insertions, 47.6% of the mutations were nucleotide deletions, and 4.8% of the mutations were simultaneous nucleotide deletions and insertions (Figure 3b). Of the insertion mutations, 100% were 1‐bp insertions, with a marked preference for C (70.0%, 21/30) nucleotide inserts over A (20.0%, 6/30), T (10.0%, 3/30) and G (0, 0/30) nucleotide inserts (Figure 3b), which was inconsistent with previous reports in rice, camelina and citrus, in which most 1‐bp insertions were A or T (Jia *et al*., 2017; Jiang *et al*., 2017; Ma *et al*., 2015b; Zhang *et al*., 2014). Most deletion mutations (63.3%, 19/30) were short (<10 bp) deletions, and the remaining 36.7% of the mutations (11/30) were longer deletions ranging from 10 bp to 91 bp (Figure 3b). Overall, approximately just over half (57.1%) of all mutations changed by only 1 bp. Expectedly, all 1‐bp indels occurred immediately upstream of the DSB position at the fourth base from the PAM site.

### Isolation of stable edited *BnCLV3* lines without T‐DNA at generation T_2_

**Figure 4:**
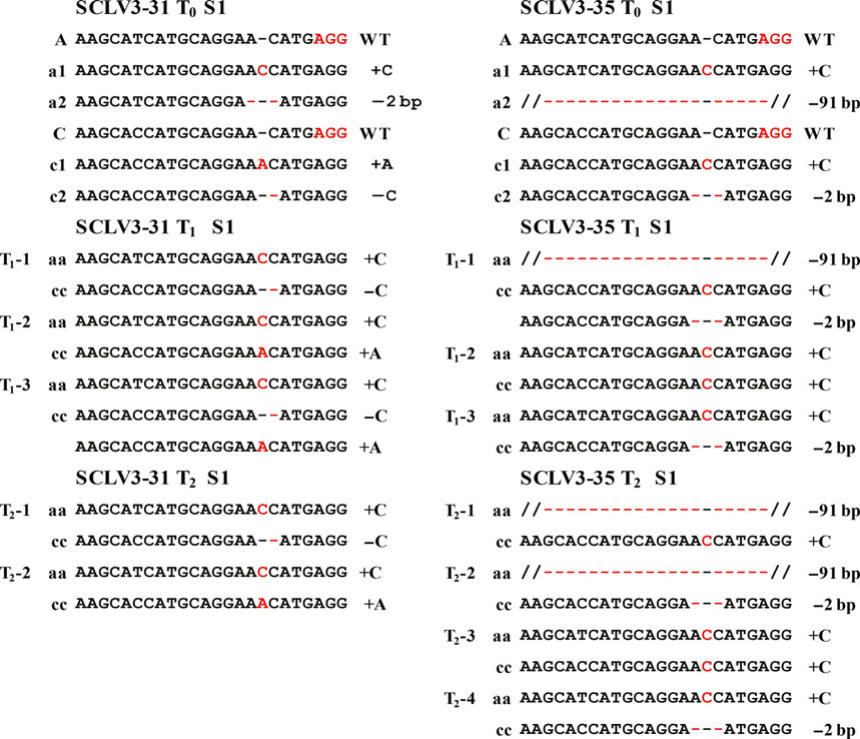
Germ‐line transmission of CRISPR/Cas9‐induced mutations at the S1 target site of SCLV3 from the T_0_ generation to the T_2_ generation. CRISPR/Cas9‐induced insertions and deletions are indicated by red font and red hyphens, respectively. On the left, A and C and the WT allele of the *BnA4*.*CLV3* and *BnC4*.*CLV3* copies, respectively; a# and c# show the mutant allele numbers. ‘−’ and ‘+’ indicate the deletion and insertion of the indicated number of nucleotides, respectively. [Colour figure can be viewed at http://wileyonlinelibrary.com]

To obtain stable mutant lines, 11 T_0_ lines carrying mutations in *BnCVL3* were self‐pollinated, and individual T_1_ progeny were genotyped via a direct sequence analysis of the PCR products of the target sites. The allelic mutations in the T_0_ mutant plants were transmitted to the T_1_ generation at an average transmission rate of 82.3% (Table 2). For instance, the mutations detected in the T_1_ progenies matched the mutations observed in the corresponding SCLV3‐31 and SCVL3‐35 T_0_ lines (Figure 4). Most lines produced T_1_ plants carrying homozygous mutations at one or two *BnCLV3* loci, indicating that the mutations were stably inherited by the next generation and fixed as homozygous genotypes (Table 2). Interestingly, two new mutations that failed in 29 T_0_ lines were identified in 519 T_1_ progeny lines at the S1 site of *BnC04.CLV3* (Figure S6), indicating that the WT site was further edited at low efficiency during the growth of the transgenic plants.

**Table 2:** Molecular and genetic analysis of CRISPR/Cas9‐induced mutations in *BnCLV3* and their transmission to the T_1_ and T_2_ generations

Analysis of T0 plants			Mutation segregation in T1 plants						Mutation segregation in T2 plants					
Plant ID	Genotype of *BnCLV3*	Mutation detected (bp)^a^	No. of tested plants with editing	WT	Hetero	Homo	Mutation transmission^b^	T‐DNA free	No. of tested plants with editing	WT	Hetero	Homo	Mutation transmission	T‐DNA free
SCLV3‐3	Aa	−8	5	0 (AA)	0 (Aa)	5 (aa)	100.0%	1/35 (2.9%)	19	0 (AA)	0 (Aa)	19 (aa)	100.0%	42/137 (0.7%)
	Cc	+1, −1		2 (CC)	3 (Cc)	0 (cc)	60.0%			17 (CC)	2 (Cc)	0 (cc)	10.5%	
SCLV3‐6	Aa	−1, +1	4	1 (AA)	2 (Aa)	1 (aa)	75.0%	5/18 (11.1%)	6	2 (AA)	3 (Aa)	1 (aa)	66.7%	9/9 (100.0%)
	Cc	−3		1 (CC)	2 (Cc)	1 (cc)	75.0%			0 (CC)	0 (Cc)	6 (cc)	100.0%	
SCLV3‐16	Aa	+1, −11	3	0 (AA)	0 (Aa)	3 (aa)	100.0%	0/34 (0%)	20	0 (AA)	0 (Aa)	20 (aa)	100.0%	2/65 (3.1%)
	Cc	+1		0 (CC)	3 (Cc)	0 (cc)	100.0%			7 (CC)	13 (Cc)	0 (cc)	65.0%	
SCLV3‐27	Aa	+1, −10, −39/+83	4	0 (AA)	2 (Aa)	2 (aa)	100.0%	12/30 (40.0%)	10	0 (AA)	0 (Aa)	10 (aa)	100.0%	23/31 (74.2%)
	Cc	+1		4 (CC)	0 (Cc)	0 (cc)	0.0%			10 (CC)	0 (Cc)	0 (cc)	0.0%	
SCLV3‐31	aa	+1, −2	4	0 (AA)	0 (Aa)	4 (aa)	100.0%	9/35 (20.0%)	5	0 (AA)	0 (Aa)	5 (aa)	100.0%	7/20 (35.0%)
	cc	+1, −1		0 (CC)	0 (Cc)	4 (cc)	100.0%			0 (CC)	0 (Cc)	5 (cc)	100.0%	
SCLV3‐35	aa	+1, −91	8	0 (AA)	0 (Aa)	8 (aa)	100.0%	2/36 (5.5%)	5	0 (AA)	0 (Aa)	5 (aa)	100.0%	3/15 (20.0%)
	cc	+1, −2		0 (CC)	0 (Cc)	8 (cc)	100.0%			0 (CC)	0 (Cc)	5 (cc)	100.0%	
SCLV3‐167	Aa	+1	2	2 (AA)	0 (Aa)	0 (aa)	0.0%	2/9 (22.2%)	9	9 (AA)	0 (Aa)	0 (aa)	0.0%	8/41 (19.5%)
	Cc	−26		0 (CC)	0 (Cc)	2 (cc)	100.0%			0 (CC)	0 (Cc)	9 (cc)	100.0%	
SCLV3‐184	aa	+1	5	0 (AA)	0 (Aa)	5 (aa)	100.0%	2/35 (5.7%)	14	0 (AA)	0 (Aa)	14 (aa)	100.0%	5/84 (6.0%)
	Cc	+1		0 (CC)	2 (Cc)	3 (cc)	100.0%			4 (CC)	3 (Cc)	7(cc)	71.4%	
UCLV3‐169	aa	+1, +1	2	0 (AA)	0 (Aa)	2 (aa)	100.0%	3/11 (18.2%)	6	0 (AA)	0 (Aa)	6 (aa)	100.0%	0/15 (0%)
	Cc	−6		0 (CC)	1 (Cc)	1 (cc)	100.0%			(CC)	3 (Cc)	3 (cc)	100.0%	
UCLV3‐29	Aa	−9	2	0 (AA)	0 (Aa)	2 (aa)	100.0%	9/18 (50.0%)	2	0 (AA)	0 (Aa)	2 (aa)	100.0%	11/11 100.0%)
	Cc	+1		1 (CC)	1 (Cc)	0 (cc)	50.0%			2 (CC)	0 (Cc)	0 (cc)	0.0%	
UCLV3‐214	aa	+1/−6, −1, −6	2	0 (AA)	0 (Aa)	2 (aa)	100.0%	4/18 (22.2%)	2	0 (AA)	0 (Aa)	2 (aa)	100.0%	7/7 (100.0%)
	Cc	+1		1 (CC)	1 (Cc)	0 (cc)	50.0%			2 (CC)	0 (Cc)	0 (cc)	0.0%	

Hetero, heterozygous; Homo, homozygous.

a ‘−’ and ‘+’ indicate the deletion and insertion of the indicated number of nucleotides, respectively; ‘−/+’ indicates the simultaneous deletion and insertion of the indicated number of nucleotides; ‘+,…’ indicates multiple types of insertions or deletions occurring in different mutation events at the same target site.

b Based on the number of plants carrying the observed mutation over the total number of plants tested.

We further analysed the transmission of the mutations from these T_1_ plants to their T_2_ offspring. The allelic mutations in the T_1_ mutant plants were transmitted to the T_2_ generation at an average transmission rate of 73.4% (Table 2). Consistently, the mutations detected in the T_2_ progeny lines matched the mutations observed in the corresponding SCLV3‐31 and SCVL3‐35 T_0_ and T_1_ lines (Figure 4). Of the 98 T_2_ plants that were sequenced, 84 (85.7%) and 35 (35.7%) plants had homozygous mutations at *BnA04.CLV3* and *BnC04.CLV3*, respectively (Table 2). Altogether, the T_1_ and T_2_ sequence data from 11 T_0_ lines provided strong evidence for stable germ‐line transmission of Cas9‐induced mutations in rapeseed.

To investigate the potential for achieving targeted modifications without incorporating foreign DNA into the rapeseed genome, we performed PCR assays of the T_1_ and T_2_ plants. The *NPT II* gene was not detected in 49 of 279 (17.6%) T_1_ plants and 117 of 435 (26.9%) T_2_ plants originating from 11 independent T_0_ lines (Table 2). Altogether, a variety of *BnCLV3* single and double homozygous T‐DNA‐free mutants were obtained in the T_2_ generation (Table S8). Therefore, T‐DNA‐free plants carrying the desired gene modifications could be acquired through genetic segregation in rapeseed.

### Multilocular phenotype can be recovered by knockout mutations of both copies of each *BnCLV* gene

Because all 1‐bp indels and most other indels change the reading frame of the gene, most mutations generated in the *BnCLV3* genes are predicted to lead to gene knockouts. Expectedly, eight of the 494 T_0_‐positive transgenic plants showed a visible knockout phenotype with multilocular silique. Three of these plants (i.e. SCLV3‐31, SCLV3‐35 and SCLV3‐73) were sequenced, and all the *BnCLV3* loci in these plants were devoid of any WT alleles at the S1 target site, showing either putative bi‐allelic mutations or chimeric mutations (Figure 3a; Table S7). Thus, the CRISPR/Cas9 system can efficiently generate targeted mutations in the rapeseed genome.

To obtain a stable knockout phenotype of *BnCLV1*, three T_0_ mutants were self‐pollinated and produced T_1_ progeny carrying various mutations at both *BnCLV1* loci (Table 3). Expectedly, six of these plants could produce multilocular siliques with two to four locules, and the percentage of multilocular siliques ranged from 2.1% to 50.0% (Table 3). Sequencing analysis revealed that all these T_1_ plants have homozygous mutations in both copies of the *BnCLV1* genes, which are predicted to lead to frameshift mutations that most likely result in nonfunctional proteins. As a control, the mutant SCLV1‐13‐4, with a 1‐bp homozygous insertion in *BnA07.CLV1*, showed a phenotype similar to WT (Table 3). Both SCLV1‐21‐12 and SCLV1‐22‐1, with frameshift mutations in *BnC06.CLV1* and deletions in multiples of three nucleotides resulting the deletion of several amino acids in *BnA07.CLV1*, also showed a phenotype similar to WT (Table 3). Thus, both copies of the *BnCLV1* gene function redundantly in multilocular trait development.

**Table 3:** Phenotypic and genotypic analysis of *BnCLV1* and *BnCLV2* mutants and their transmission to T_1_ generation

Line	Generation	Multilocular siliques%^a^	*BnA07.CLV1*				*BnC06.CLV1*			
			S3^b^	S4	S5	S6	S3	S4	S5	S6
SCLV1‐13	T_0_	/	+A, Homo	WT	WT	WT	WT	WT	WT	WT
SCLV1‐13‐4	T_1_	0.0	+A, Homo	WT	WT	WT	WT	WT	WT	WT
SCLV1‐21	T_0_	/	Chimeric	/	/	/	−11 bp, Homo	WT	WT	WT
SCLV1‐21‐3	T_1_	3.1	−37 bp, Homo	WT	WT	WT	−11 bp, Homo	WT	WT	WT
SCLV1‐21‐24	T_1_	40.7	−37 bp, Homo	WT	WT	WT	−11 bp, Homo	WT	WT	WT
SCLV1‐21‐6	T_1_	2.1	−37 bp, +1 bp	WT	WT	WT	−11 bp, Homo	WT	WT	WT
SCLV1‐21‐2	T_1_	18.2	−37 bp, −15 bp, +1 bp	WT	WT	WT	−11 bp, Homo	WT	WT	WT
SCLV1‐21‐5	T_1_	50.0	−37 bp, −15 bp, +1 bp	WT	WT	WT	−11 bp, Homo	WT	WT	WT
SCLV1‐21‐25	T_1_	4.0	−37 bp, −15 bp, +1 bp	WT	WT	WT	−11 bp, Homo	WT	WT	WT
SCLV1‐21‐12	T_1_	0.0	−15 bp, Homo	WT	WT	WT	−11 bp, Homo	WT	WT	WT
SCLV1‐22	T_0_	/	Hetero	/	/	/	+G, Homo	WT	WT	WT
SCLV1‐22‐1	T_1_	0.0	−27 bp, Homo	WT	WT	WT	+G, Homo	WT	WT	WT
			*BnA02.CLV2*				*BnC02.CLV2*			
			S7	S8	S9	S10	S7	S8	S9	S10
SCLV2‐11	T_0_	/	−A, Homo	WT	WT	WT	Hetero	/	WT	WT
SCLV2‐11‐2	T_1_	0.0	−A, Homo	WT	WT	WT	−A, −3 bp	WT	WT	WT
SCLV2‐12	T_0_	/	Chimeric	/	WT	WT	Hetero	/	/	Hetero
SCLV2‐12‐9	T_1_	10.0	+A, Homo	+C, Homo	WT	WT	−A, Homo	WT	WT	WT
SCLV2‐12‐10	T_1_	44.0	+G, −3 bp	+C, Homo	WT	WT	−A, Homo	WT	WT	+T, Hetero
SCLV2‐12‐11	T_1_	10.5	+G, −3 bp	+C, Homo	WT	WT	−A, Homo	WT	WT	WT
SCLV2‐24	T_0_	/	WT	WT	WT	WT	Hetero	/	WT	WT
SCLV2‐24‐3	T1	0.0	WT	WT	WT	WT	+A, Homo	WT	WT	WT
SCLV2‐24‐4	T1	0.0	WT	WT	WT	WT	+A, Homo	WT	WT	WT

See footnotes for Table 2.

Similarly, three T_0_ editing lines were self‐pollinated and produced T_1_ progeny carrying various mutations at different target sites at the *BnCLV2* loci (Table 3). Consistently, three T_1_ plants originating from SCLV2‐12 could produce multilocular siliques with two to four locules, reflecting frameshift mutations in both *BnCLV2* copies (Table 3). As a control, the mutants SCLV2‐24‐3 and SCLV2‐24‐4, each with a 1‐bp homozygous insertion in *BnC02.CLV2*, showed a phenotype similar to WT (Table 3). A T1 line (i.e. SCLV2‐11‐2) with a 1‐bp homozygous deletion in *BnA02.CLV2* and heterozygous mutations in *BnC02.CLV2* showed a phenotype similar to WT (Table 3). Thus, the multilocular trait is also controlled by both *BnCLV2* copies with redundant functions in rapeseed.

### The number of leaves, NSS and the SW were significantly increased in the *BnCLV3* mutants

**Figure 5:**
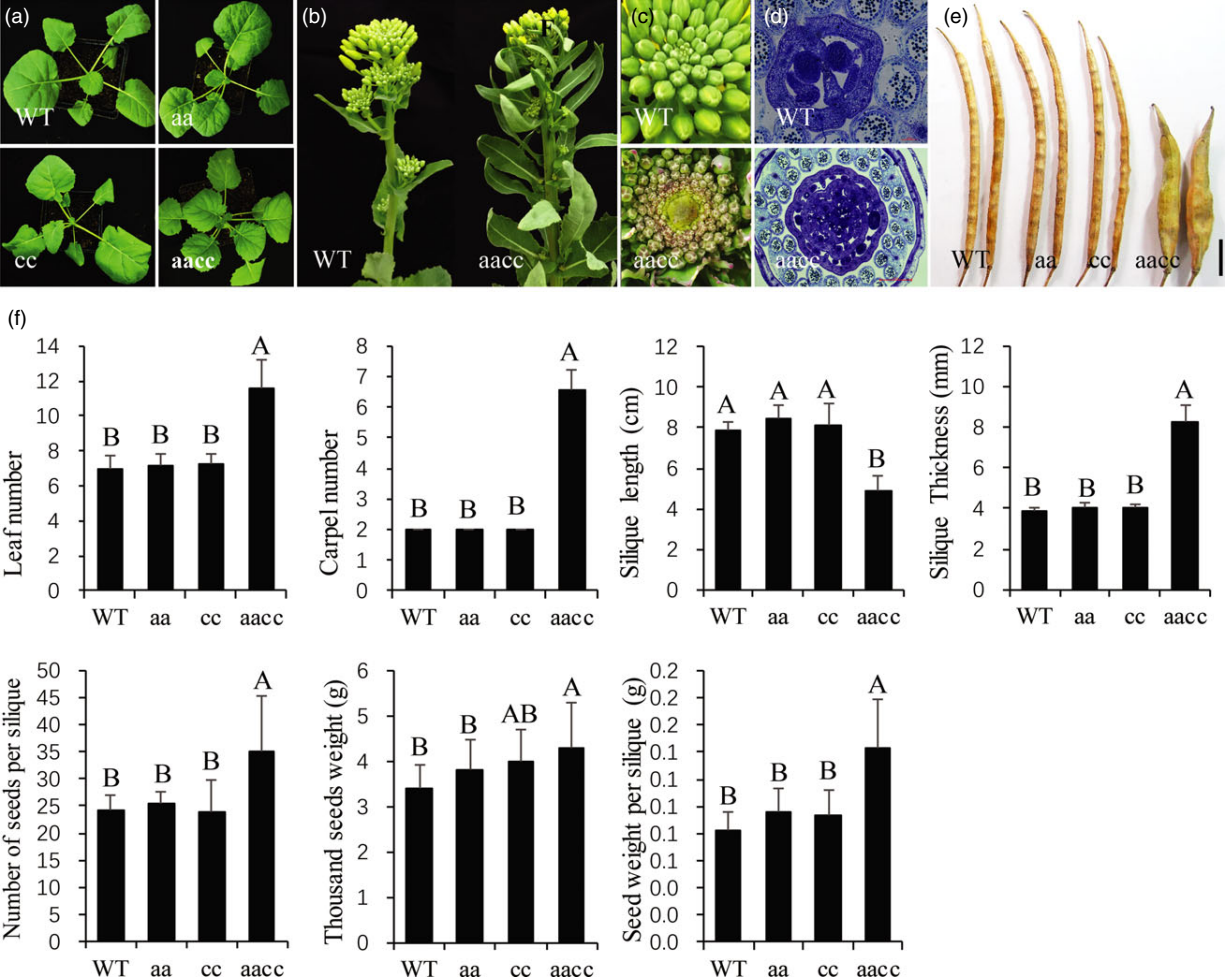
Phenotypes of the *BnCLV3* mutants. (a) Leaf numbers of 30‐day‐old seedlings in WT, single and double homozygous mutants of *BnCLV3*. (b, c) The inflorescences (b) and SAM (c) in the WT and a double homozygous mutant of *BnCLV3*. (d) Cross sections of gynoecia in the WT and a double homozygous mutant of *BnCLV3* at stages 9–10. (e) Siliques in the WT, single and double homozygous mutants of *BnCLV3*. Bar = 1 cm. (f) Statistical analysis of the leaf number, carpel number, silique length, silique thickness, NSS, thousand seeds weight and SW per silique in the WT and single and double homozygous mutants of *BnCLV3*. The data and error bars represent the mean ± SD (*n* ≥ 15 plants for each genotype). Upper‐case letters indicate a significant difference at the 0.01 probability level. aa, homozygous mutation of *BnA04*.*CLV3*; cc, homozygous mutation of *BnC04*.*CLV3*; aacc, double homozygous mutation of *BnA04*.*CLV3* and *BnC04*.*CLV3*. [Colour figure can be viewed at http://wileyonlinelibrary.com]

To characterize the phenotype of the *BnCLV3* mutants, all homozygous mutant T_2_ lines with different frameshift mutations (Table S8) were grown in the field. The leaf number in the 30‐d‐old seedlings in the double mutants was significantly higher (*P *<* *0.01) than in the WT control plants, whereas this trait in the two single mutants of *BnCLV3* was comparable to WT control (Figure 5a,f). The leaf numbers and the size of the SAMs of inflorescences were also dramatically higher in the double mutants than in the WT control (Figure 5b and c). Consistent with this finding, the number of the four floral organs was significantly higher (*P *<* *0.01) only in the double mutants (Figures 5d,f; S7). All the siliques in the double mutants were multilocular, ranging from 5.0 to 7.9 with a mean ± SD value of 6.6 ± 0.7 locules per silique, although the WT and both single mutants were bilocular siliques (Figure 5e,f). The multilocular siliques were shorter, rounder and thicker as reported in *B. rapa* (Fan *et al*., 2014; Figure 5e,f). The NSS and TSW were simultaneously increased to more than 10.8 and 0.92 g, compared with the WT average of 24.4 and 3.38 g, respectively; consequently, the SW per silique was increased by more than 74.4% on average, in contrast to 0.08 g in the WT (Figure 5f). Thus, *BnA04.CLV3* and *BnC04.CLV3* contribute to the increased leaf number and locule number of siliques, and the simultaneous mutation of the two homo‐alleles confers a multilocular trait with high‐yield potential. Therefore, the double mutants of *BnCLV3* generated in the present study might provide excellent starting materials for high‐yield breeding in rapeseed.

### Off‐target activity of CRISPR/Cas9 in T_0_ transgenic *B. napus* plants

To determine whether off‐targeting occurred in the present study, we searched the *B. napus* genome for putative off‐target sites with a high homology to the five sgRNAs that detected on‐target mutations according to the CRISPR‐P program (Lei *et al*., 2014). These potential off‐target sites and their related genome positions are listed in Table S9. There were 17, 18, 11, 4 and 7 putative off‐target sites for S1, S3, S4, S7 and S8, respectively (Table 4).

**Table 4:** Detection of potential off‐target effects for each sgRNA target site in T_0_ mutated plants

On‐target site	Binary vector	No. of plants for sequencing	Putative off‐target sites	Off‐target editing
S1	SCLV3 + UCLV3	47	17	No
S3	SCLV1	22	18	No
S4	SCLV1	22	11	No
S7	SCLV2	45	4	No
S8	SCLV2	45	7	No

High‐throughput sequencing of the PCR products of these 57 potential sites from many T_0_ mutated plants showed no mutations (Tables 4; S9), suggesting that the off‐target effect is negligible when the sgRNA specificity is considered well according to the genome sequence. Thus, the CRISPR/Cas9 system has a high specificity for targeted mutagenesis in *B. napus*.

### Genome editing of cultivars by crossing genome‐edited lines with other cultivars

**Figure 6:**
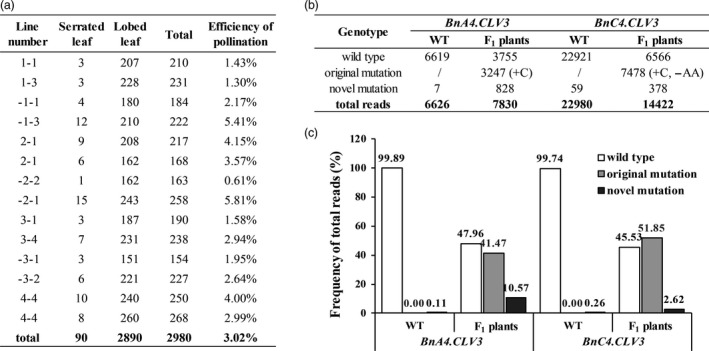
Genome editing of cultivars by crossing Cas9‐sgRNA lines. (a) Selection of the natural outcrossing plants from open‐pollination progeny of HY. Plants with serrated leaves are hybrids of HY and the double homozygous mutant SCLV3‐35 for the incompletely dominant lobed‐leaf trait. (b) The read numbers at the S1 site of *BnCLV3* in mixed genomic DNA from F_1_ hybrid plants with T‐DNA are shown, and WT (HY) was included as a control. (c) Frequency of different genotype reads. Original mutant reads, reads with the same mutations detected in SCLV3‐35; novel mutant, reads with different mutations detected in SCLV3‐35.

Because few cultivars are amenable to transformation and introducing recessive alleles by crossing is tedious, we explored the efficiency of the horizontal transfer of the Cas9/gRNA cassettes using open pollination. The *B. napus* cultivar HY, which has a specific lobe‐leaf trait, was grown near the T_2_ SCLV3‐35 lines (homozygous mutation in *BnA04.CLV3* and bi‐allelic mutation in *BnC04.CLV3* at the S1 site) in an isolated area of the field to control pollination. Open‐pollination seeds from HY were germinated, and seedlings with different leaf shapes from HY, which is an indicator of hybridization with SCLV3‐35 for the incompletely dominant lobed‐leaf trait, were selected for further genotyping. In total, 90 of the 2980 seedlings were selected using this method for an approximately 3.02% natural outcrossing rate (Figure 6a). Of these 90 seedlings, 68 seedlings carried the transgene insertion transmitted from the SCLV3‐35 progeny. Mixed genomic DNA from these plants was used as the template for the amplification of the S1 site using *BnCLV3*‐specific primers. The products were purified to generate a DNA library for high‐throughput sequencing. WT plants (HY) were also included in the genome resequencing and subsequent analysis as controls. The reads from the S1 site of *BnCLV3* were aligned to the WT DNA sequences to detect mutations, and approximately 1 WT:1 mutant reads were observed at the S1 site of each copy of *BnCLV3* (Figure 6b,c). Almost all mutated reads matched the reads observed in the corresponding parental SCLV3‐35 line (original mutants, Figure 6b,c). Interestingly, various novel mutations were detected at the S1 site of *BnA04.CLV3* in these progeny with 10.57% efficiency, which clearly showed that all novel mutations occurred in the HY allele based on the polymorphism between the HY and SCLV3‐35 lines in the amplicon fragment (Figure 6b,c; Table S10). This result indicated that the WT allele (HY) was further edited with a low efficiency in the presence of CRISPR‐Cas9 (Figure 6b,c; Table S10). Various novel mutations were also detected at the S1 site of *BnC04.CLV3* in these progeny with 2.62% efficiency. However, it could not be determined in which allele the novel mutation had occurred because there were no polymorphisms between the HY and SCLV3‐35 lines in the amplicon fragment of *BnC4.CLV3* (Table S10). Additionally, a few unexpected reads with novel mutations in the WT DNA were also observed, which might reflect primer mismatches during PCR because of the much lower frequency than that of the HY progeny (Figure 6b,c).

## Discussion

In this study, we used RNA‐guided Cas9 to induce targeted mutations in rapeseed and report the stable transmission of mutations across generations. P_35s_ resulted in better Cas9 protein expression and a higher efficiency of mutagenesis in rapeseed than P_ubi_, which was inconsistent with a previous report by Ma *et al*. (2015b), who recommended P_ubi_ rather than P_35s_ for Cas9 protein expression in dicot plants. A visible multilocular silique can be recovered after knocking out all copies of each *BnCLV* gene in rapeseed, which is similar to observations in *Arabidopsis* and exemplifies the use of RNA‐guided Cas9 to target important traits in *Brassica* crops based on knowledge regarding gene function from model plants. We assayed 57 potential off‐target loci, and none of these genes showed evidence of a CRISPR/Cas9 system‐induced mutation, indicating that well‐designed specific sgRNAs do not target undesired sites. We obtained a variety of transgene‐free *B. napus* plants with homozygous mutations in the target gene, supporting the potential for further biotechnological applications.

Several crucial factors, including the expression levels of Cas9 and sgRNA, GC% content, targeting context and secondary structure of the target sgRNAs, may influence sgRNA efficacy in plants (Ma *et al*., 2015b; Makarova *et al*., 2011; Wang *et al*., 2014). In the present study, the mutagenesis efficiency had a wide range – from 0% to 48.7% in T_0_ for the ten sgRNAs (Table 1). To determine the parameters affecting the Cas9 targeting efficiency in rapeseed, we analysed the GC% content and secondary structures of all target sgRNAs, but we did not detect any association with their respective editing efficiency (Table 1). We further detected the expression of Cas9p and sgRNAs in T_0_‐positive transgenic lines with targeting of the S3‐S6 sites of the *BnCLV1* genes. These gRNAs targeting the same gene exhibited dramatically variable genome‐editing efficiencies, such as 46.5% in S3, 5.0% in S4 and 0% in S5 and S6. The overall levels of Cas9 and sgRNAs in these plants were similar (Figure S8), although the S3 and S4 targets were edited in six plants, and S5 and S6 failed to show any editing. The expression levels of Cas9 and sgRNAs might not be the limiting factors of genome editing in *B. napus* T_0_ plants. In summary, the variations in the mutagenesis efficiency in different sgRNAs could most likely reflect differences in the nucleotide composition of the sgRNAs. Although prediction algorithms have been developed to evaluate the guide activity (Haeussler *et al*., 2016; Lei *et al*., 2014), the accuracy of these algorithms in different crops, such as *B. napus*, requires additional studies.

Plant genome‐editing techniques largely depend on plant genetic transformation. Compared with other crops, the transformation efficiency in most *B. napus* cultivars remains low. For instance, Braatz *et al*. (2017) reported site‐directed mutagenesis using the Cas9/sgRNA system in rapeseed using the spring cultivar Haydn as the transformation recipient and showed that only one transgenic plant regenerated at the first generation for a transformation rate of 0.9%. Thus, the application of CRISPR/Cas9‐induced mutations in rapeseed is likely limited in certain currently cultivated plants. In the present study, the pure *B. napus* line J9707 was a good transformation recipient at a transformation rate ranging from 66.7% to 92.5%. Thus, we hypothesize that crossing Cas9‐gRNA lines generated from a suitable transformation recipient with other cultivars, together with marker‐assisted selection, is an alternative for achieving desired gene modifications.

The allotetraploid *B. napus* contains two distinct but closely related homologous subgenomes. Consequently, certain important traits are controlled by several gene copies with redundant functions in both subgenomes in rapeseed. For instance, the multilocular trait in the present study was controlled by both copies of each *BnCLV* gene, and only double homozygous mutants showed this knockout phenotype. To date, no spontaneous or induced multilocular mutants have been reported in *B. napus*, likely reflecting its allotetraploid nature and the extremely low probability of double mutations in the same plant. Thus, the simultaneous alteration of multiple gene copies by CRISPR/Cas9 mutagenesis has great potential in revealing gene function and generating agronomically important mutations in crops. In a previous study, a single‐nucleotide mutation in a *BrCLV3* gene homologue resulted in a weak mutation phenotype that produced siliques with 3‐4 locules and an increased NSS without a reduction in TSW (Fan *et al*., 2014). In the present study, the double homozygous mutants of the *BnCLV3* genes likely exhibited a full *clv3* loss‐of‐function phenotype, with siliques harbouring 5.0–7.9 locules, and significantly increased NSS and TSW (Figure 5d–f). Consequently, the SW per silique in the double mutants of *BnCLV3* increased by more than 74.4% on average compared with that of WT (Figure 5f). In addition, an increase in the leaf number was also not observed in the *clv3* mutants in *B. rapa* and *Arabidopsis*. Thus, the double mutants of *BnCLV3* generated in the present study might provide excellent starting materials for further high‐yield breeding in rapeseed. In *B. juncea*, a mutation in the *CLV1* gene homologue on the B genome exhibited four valves stably in trilocular siliques (Xu *et al*., 2017). Compared with the mutation phenotype of the same gene homologue in *B. juncea*, the phenotype of the double mutants of *BnCLV1* generated in the present study was instable; that is, they produced a variable valve numbers in the siliques of the same plant and a lower percentage of multilocular siliques per plant (Table 3). The multilocular silique phenotype of the double mutants of *BnCLV2* was also instable as that of *BnCLV1* (Table 3). Thus, the genetic control of multilocular siliques was more complicated in *B. napus*. Based on the prevailing model for *CLV* signalling in *Arabidopsis*, CLV3 is recognized by at least three functionally redundant receptors, including CLV1, the CLV2/CRN complex and RPK2 (Clark *et al*., 1997; Jeong *et al*., 1999; Kinoshita *et al*., 2010). Thus, it is understandable that the mutation phenotypes of *BnCLV1* and *BnCLV2* are less stable than *BnCLV3*. The large and specific mutant diversity of *BnCLV1* and *BnCLV2* generated in the present study provided a valuable resource for further genetic studies in rapeseed.

In conclusion, our work presents a successful example to utilize CRISPR/Cas9‐induced mutations for revealing gene functions in polyploid species and also provides agronomically important crop mutations.

## Experimental procedures

### Plant material

The semi‐winter *B. napus* pure line J9707 was used as the transformation receptor in this study. Another semi‐winter *B. napus* cv. HY, which has a lobed‐leaf phenotype, was used for the pollination testing along with the genome‐edited line SCLV3‐35. All seeds were obtained from the National Engineering Research Centre of Rapeseed, Wuhan, China.

### CRISPR/Cas9 target locus selection and construct assembly

Sequence‐specific sgRNAs were designed using the web‐based tool CRISPR‐P (http://cbi.hzau.edu.cn/cgi-bin/CRISPR). Two or four output target sites were selected for each target gene based on their location in the gene, GC% content and putative off‐targets (Figure 1a,c,d). These targets were assessed using PCR and Sanger sequencing in J9707 to ensure that no polymorphisms existed between the sgRNAs and the corresponding target sequences.

The binary pYLCRIPSR/Cas9 multiplex genome targeting vector system, which was provided by Prof. Yaoguang Liu (South China Agriculture University), included pYLCRISPR/Cas9P_ubi_‐H and pYLCRISPR/Cas9P_35S_‐H, in which Cas9p is driven by the maize ubiquitin promoter (P_ubi_) and the cauliflower mosaic virus 35S promoter (P_35S_), and four plasmids with sgRNA cassettes driven by the promoters of AtU3b, AtU3d, AtU6‐1 and AtU6‐29; this system was used for construct assembly according to a method previously described by Ma *et al*. (2015b). The oligos used to construct the sgRNA vectors are listed in Table S1. The resulting constructs contained a Cas9p expression cassette, sgRNA expression cassettes with target sequences and a hygromycin resistance cassette (Figure 1b,e).

### Agrobacterium‐mediated transformation of rapeseed

The Cas9/sgRNA‐expressing binary vectors were transformed into J9707 via the *Agrobacterium tumefaciens*‐mediated hypocotyl method (Zhou *et al*., 2002). Regenerated seedlings were selected according to their hygromycin resistance, further cultivated in a growth chamber under a 14‐h light/10‐h dark cycle at 25 °C and transferred to a field during the rapeseed growing season.

### Identification of mutant transgenic plants

The presence of a T‐DNA construct was assessed by PCR using the *NPT II* gene‐specific primers 35S‐3/HPT F (Table S1).

PCR was performed to amplify the genomic region surrounding the CRISPR target sites using specific primers (Table S1), and the mutations were screened using the PAGE method previously described by Zhu *et al*. (2014). Briefly, the PCR products were denatured at 90°C for 5 min, followed by cooling to room temperature for renaturing. The renatured PCR products were separated using native PAGE. To confirm the results of the PAGE‐based genotyping, the PCR fragments were directly sequenced or cloned into the pEASY‐T vector (TransGen Biotech, Beijing, China) and subsequently sequenced using a Sanger method to identify the mutations. The sequences were compared to WT sequences to detect the presence of indels. The sequencing chromatograms were also examined to identify overlapping traces in the region surrounding the PAM, which are indicative of the presence of mutations. The bi‐allelic and heterozygous mutations were decoded using the degenerate sequence decoding (DSD) method (Ma *et al*., 2015a).

### Phenotyping of *B. napus* transgenic lines

The WT and homozygous T_2_ mutant lines were grown during the winter‐type oilseed rape growing season on an experimental farm at Huazhong Agriculture University, Wuhan, China. The leaf number of 30‐day‐old seedlings was measured based on at least 30 plants per genotype. At the flowering stage, at least 30 flowers were randomly selected from each plant to count the floral organ number, including the number of sepals, petals, stamens and carpels. All mature siliques were collected from each plant to determine the percentage of multilocular siliques; thirty siliques were randomly sampled from each plant and used to measure the silique length, silique thickness and seed number per silique. The cleaned seeds were air‐dried for at least 4 weeks. The SW of each plant was measured based on 100 fully developed seeds with three replicates.

### Analysis of potential off‐targets

The potential off‐target sites were identified using CRISPR‐P (http://cbi.hzau.edu.cn/cgi-bin/CRISPR). An approximately 300‐bp DNA sequence covering each off‐target site was amplified by PCR. The primers are listed in Table S1. For each target gene, mixed genomic DNA from T_0_ editing plants was used as the template, and WT DNA was included as a control. All PCR products were purified and mixed in equal amounts (50 ng for each) as one sample. The DNA library construction, sequencing using the Illumina HiSeq 3000 system and data analysis were conducted according to the methods previously described by Wang *et al*. (2017). The independent sequence reads of each off‐target site were aligned to the genomic WT sequence, which covered each off‐target site as a reference.

### Southern blotting analysis

Genomic DNA was isolated from the young leaves by the cetyltrimethylammonium bromide method. A total of 30 μg of genomic DNA was digested with *Hin*dIII and then separated on a 0.8% agarose gel. After electrophoresis, the digested DNA was transferred onto a nylon membrane. For hybridization, a 293‐bp 3′‐terminus conserved *BnCLV3* sequence was used as a probe.

## Supporting information


**Figure S1** Determination of *BnCLV3* gene copy number by southern blotting analysis in J9707 and Darmor‐bzh.
**Figure S2** Sequence alignment of two *BnCLV3* gene copies.
**Figure S3** Sequence alignment of the two *BnCLV1* gene copies.
**Figure S4** Sequence alignment of the two *BnCLV2* gene copies.
**Figure S5** Detection of a series of mutations with different indel sizes using a PAGE‐based method.
**Figure S6** Novel mutations were detected in the T_1_ progeny with T‐DNA transmission.
**Figure S7** Variations in the floral organs in the double homozygous mutants of *BnCLV3*.
**Figure S8** Expression of *Cas9p* and sgRNAs in SCLV1. (a) The genotypes of eight T_0_ plants used for the gene expression analysis.

**Table S1** Primers used in the present study.
**Table S2** Mutations identified at the target sites in *BnCLV3* T_0_ plants using PAGE‐based screening.
**Table S3** Mutations identified at the target sites in *BnCLV1* T_0_ plants using PAGE‐based screening.
**Table S4** Mutations identified at the target sites in *BnCLV2* T_0_ plants using PAGE‐based screening.
**Table S5** Ratios of mutant genotype at the target sites in T_0_ *BnCLV1* plants.
**Table S6** Ratios of mutant genotype at the target sites in T_0_ *BnCLV2* plants.
**Table S7** Genotypes at the S1 target site in 22 T_0_ plants of *BnCLV3* by sequencing after TA cloning.
**Table S8** Variety of T‐DNA‐free *BnCLV3* T_2_ generation homozygous mutants.
**Table S9** Detection of potential off‐target effects at each sgRNA target site.
**Table S10** Alignment of novel mutation sequences in the transmission analysis of CRISPR/Cas9‐induced mutations.
