# Supplementary material for: Precise editing of CLAVATA genes in Brassica napus L. regulates multilocular silique development
Source: Plant Biotechnol J. 2018 Jan 19;16(7):1322–35. doi: 10.1111/pbi.12872 (PMC5999189; doi:10.1111/pbi.12872)
Supplement: Supplementary file 1 — Figure S1 Determination of BnCLV3 gene copy number by southern blotting analysis in J9707 and Darmor‐bzh. Figure S2 Sequence alignment of two BnCLV3 gene copies. Figure S3 Sequence alignment of the two BnCLV1 gene copies. Figure S4 Sequence alignment of the two BnCLV2 gene copies. Figure S5 Detection of a series of mutations with different indel sizes using a PAGE‐based method. Figure S6 Novel mutations were detected in the T1 progeny with T‐DNA transmission. Figure S7 Variations in the floral organs in the double homozygous mutants of BnCLV3. Figure S8 Expression of Cas9p and sgRNAs in SCLV1. (a) The genotypes of eight T0 plants used for the gene expression analysis. [file PBI-16-1322-s001.docx]

**Supplementary Figures**

**
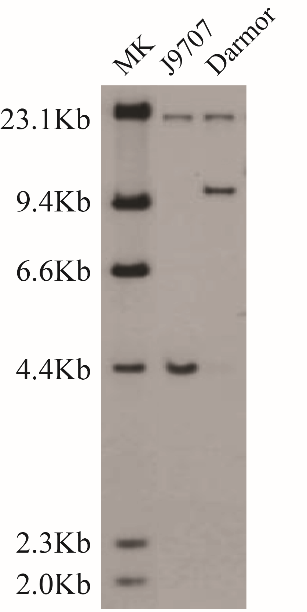
**

**Fig. S1** Determination of *BnCLV3* gene copy number by southern blotting analysis in J9707 and Darmor-bzh. The genomic DNA was digested with *Hin*dIII before electrophoresis. MK, DNA marker.

**(a)**

S1

***BnA4.CLV3*** ATGGATTCGA GGACTCTGGT GCTACTGCTG CTCTTTTG**CC TCATGTTCCT** 50

***BnC4.CLV3*** ATGGATTCGA GGACTCTGGT GCTACTGCTG CTCTTTTG**CC TCATGTTCCT** 50

***Clustal***  ********** ********** ********** ********** ********** 50

***BnA4.CLV3*** **GCATGATGCT T**CTGGTGTCT TTCCCTTTCT CTCTCTTCTA TATATATATA 100

***BnC4.CLV3*** **GCATGGTGCT T**CTGGTCTCT TCCCCTTTCC CTCTCTTCTG TATATATAT- 99

***Clustal***  ***** **** ****** *** * ******* ********* ********* 94

***BnA4.CLV3*** TATATAAATA TATCGCTCAT GTTCACATAT TCTTACAATT ACAATGTTTT 150

***BnC4.CLV3*** ---------- ---CGCTCAT GTTCACATAT CCTTACAATT ACAATGTTTT 136

***Clustal***  ******* ********** ********* ********** 130

***BnA4.CLV3*** CTACAGATAT CACTCACGCC AATGCCAATG TTCATGCACT TCCCATTCGC 200

***BnC4.CLV3*** CTACAGATAT CACTCACGCC AATGCCAATG TTCATGCACT TCCCATTCGC 186

***Clustal***  ********** ********** ********** ********** ********** 180

***BnA4.CLV3*** AAGGTTTGCT TCTCAACATT TACACACACA CGCACATA-- --TATATATA 246

***BnC4.CLV3*** AAGGTTTGCT TCTCAACATT TACACACACA CACACACACA CATATATATA 236

***Clustal***  ********** ********** ********** * **** * ******** 224

***BnA4.CLV3*** TATCATTTCT GTATTGTTAT AAACACGTAC GCGCACGCGG ACATCTATCT 296

***BnC4.CLV3*** TATCATTTCT GTATTGTTAT AAACACGTAC GCGCACGCGG ACATCTATCT 286

***Clustal***  ********** ********** ********** ********** ********** 274

***BnA4.CLV3*** CGTGTTTGGA ATATACTACA GTGTGCATGT TTTTGCTATG ATACATACTA 346

***BnC4.CLV3*** CGTGTTTGGA ATATACTACA GTGTGCATGT TTTTGCTATG ATACATACTA 336

***Clustal***  ********** ********** ********** ********** ********** 324

***BnA4.CLV3*** AAATGGTGAT TGATTTGGCA GATGATGGTA ATGAAGAAGG ATAATGAATG 396

***BnC4.CLV3*** AAATGGTGAT TGATTTGGCA GATGATGGTA ATGAAGAAGG ATAATGAATG 386

***Clustal***  ********** ********** ********** ********** ********** 374

***BnA4.CLV3*** GGGAGGAGCA AATGGAATTG AAGAAGAGAA GGAGAAGGTT TTCGGGTTAA 446

***BnC4.CLV3*** GGGAGGAGCA AATCGAATTG AAGAAGAGAA GGAGAAGGTT TTCGGGTTAA 436

***Clustal***  ********** *** ****** ********** ********** ********** 423

**S2**

***BnA4.CLV3*** ATGAAGAACT AAGGACTGT**C CCTTCAGGAC CTGACCCTTT GC**ACCATCAT 496

***BnC4.CLV3*** ATGAAGAGCT AAGGACTGT**C CCTTCAGGAC CTGACCCTTT GC**ACCATCAT 486

***Clustal***  ******* ** ********** ********** ********** ********** 472

***BnA4.CLV3*** GTGAACCCCC CAAGAAAGCC ACGAACCGAC TCTCATATCC CTTAA 541

***BnC4.CLV3*** GTGAACCCCC CAAGAAAGCC ACGAACCGAC TCTCATATCC CTTAA 531

***Clustal***  ********** ********** ********** ********** ***** 517

**(b)**

***BnA4.CLV3*** MDSRTLVLLL LFCLMFLHDA SDITHANANV HALPIRKMMV MKKDNEWGGA 50

***BnC4.CLV3*** MDSRTLVLLL LFCLMFLHGA SDITHANANV HALPIRKMMV MKKDNEWGGA 50

***Clustal*** ********** ********.* ********** ********** ********** 49

***BnA4.CLV3*** NGIEEEKEKV FGLNEELRTV PSGPDPLHHH VNPPRKPRTD SHIP 94

***BnC4.CLV3*** NRIEEEKEKV FGLNEELRTV PSGPDPLHHH VNPPRKPRTD SHIP 94

***Clustal*** * ******** ********** ********** ********** **** 92

**Fig. S2** Sequence alignment of two *BnCLV3* gene copies. (**a**) Gene sequence alignment between the *BnA04.CLV3* and *BnC04.CLV3* copies. The target sequences are shown in bold and underlined with the PAM highlighted in red. (**b**) Protein sequence alignment between the *BnA04.CLV3* and *BnC04.CLV3* copies. The black boxes highlight polymorphisms.

(**a**)

***BnA7.CLV1*** ATGGAGATGA GACTTCTGAA AACTCACCTT CTGTTTCTCC ATCTTCATTA 50

***BnC6.CLV1*** ATGGAGATGA GACTTTTGAA AACTCACCTT CTGTTTCTCC ATCTTCACTA 50

***Clustal***  ********** ***** **** ********** ********** ******* ** 48

***BnA7.CLV1*** CGTTATCTCG ATTTCGCTTC TATGTTTCTC ACCATGCCTC GCTT**CCACTG** 100

***BnC6.CLV1*** CGTTATCTCG ATTTTGCTTC TATCTTTCTC ACCATGCTTC GCTT**CCACTG** 100

***Clustal***  ********** **** ***** *** ****** ******* ** ********** 95

**S3**

***BnA7.CLV1*** **ACATGGACCA TCTCCTC**ACC CTCAAATCGT CCATGGTCGG TCCCAACGGC 150

***BnC6.CLV1*** **ACATGGACCA TCTCCTC**ACC CTCAAATCGT CCATGGTCGG CCCCAACGGC 150

***Clustal***  ********** ********** ********** ********** ********* 144

***BnA7.CLV1*** AACGGCCTCC ACGACTGGGT TCACTCCACT TCTCCCGCAG CTCACTGTTC 200

***BnC6.CLV1*** CACGGCCTCC ACGACTGGGT TCGCTCCCCT TCTCCCTCAG CTCACTGTTC 200

***Clustal***  ********* ********** ** **** ** ****** *** ********** 190

***BnA7.CLV1*** TTTCTCCGGC GTTTCATGCG ACGGAGACGC TCGTGTCATC TCCCTCAACG 250

***BnC6.CLV1*** TTTCTCCGGC GTTTCCTGCG ACGGCGACGC TCGTGTCATC TCCCTCAACG 250

***Clustal***  ********** ***** **** **** ***** ********** ********** 238

***BnA7.CLV1*** TCTCTTTCAC TCCTCTCTTC GGAACCATCT CGCCGGAGAT TGGGATGCTG 300

***BnC6.CLV1*** TCTCTTTCAC TCCTCTCTTC GGAACCATCT CCCCGGAGAT TGGGATGCTG 300

***Clustal***  ********** ********** ********** * ******** ********** 287

***BnA7.CLV1*** AACCGTCTTG TGAATCTGAC GTTAGCCGCT AATAACTTCT CCGGTATGTT 350

***BnC6.CLV1*** GACCGTCTCG TGAATCTGAC GTTAGCTGCT AATAATTTCT CCGGTATGCT 350

***Clustal***  ******* * ********** ****** *** ***** **** ******** * 332

***BnA7.CLV1*** GCCGCTGGAG ATGAAGAGTC TCACTTCTCT AAAGGTTCTC AACGTCTCCA 400

***BnC6.CLV1*** CCCGTTGGAG ATGAAGAGTC TCACTTCTCT AAAGGTTCTC AACATCTCCA 400

***Clustal***  *** ***** ********** ********** ********** *** ****** 379

**S4**

***BnA7.CLV1*** ACAACGTGAA CCTCAACGGA ACCTTCCC**CG GAGAGATTCT CACTCCCATG** 450

***BnC6.CLV1*** ACAACGTGAA CCTCAACGGA ACCTTCCC**CG GAGAGATTCT CACTCCCATG** 450

***Clustal***  ********** ********** ********** ********** ********** 429

***BnA7.CLV1*** **G**TGGACCTCG AAGTCCTCGA CGCGTACAAC AACAACTTCA CAGGCCCATT 500

***BnC6.CLV1*** **G**TCGACCTCG AAGTCCTCGA CGCGTACAAC AACAACTTCA CAGGCCCACT 500

***Clustal***  ** ******* ********** ********** ********** ******** * 477

***BnA7.CLV1*** ACCCCCGGAG ATCCCCGGGC TCAAGAAGCT GAGACACCTC TCTCTCGGAG 550

***BnC6.CLV1*** ACCGCCGGAG ATCCCCGGGC TCAAGAAGCT GAGACACCTC TCTCTCGGAG 550

***Clustal***  *** ****** ********** ********** ********** ********** 526

**S5**

***BnA7.CLV1*** GAAACTTCTT AACCGGAGAG ATC**CCAGAGA GTTACGGAGA CATCCA**AAGC 600

***BnC6.CLV1*** GAAACTTCTT AACCGGAGAA ATC**CCAGAGA GTTACGGAGA TATCCA**GAGC 600

***Clustal***  ********** ********* ********** ********** ***** *** 573

***BnA7.CLV1*** TTGGAGTATC TCGGCCTCAA CGGAGCCGGA CTCTCCGGTG AATCTCCGGC 650

***BnC6.CLV1*** TTGGAGTATC TTGGCCTCAA CGGAGCCGGA CTCTCCGGCG AATCTCCGGC 650

***Clustal***  ********** * ******** ********** ******** * ********** 621

***BnA7.CLV1*** GTTCTTGTCT CGCCTCAAGA ATCTTAAAGA AATGTACGTC GGCTACTTCA 700

***BnC6.CLV1*** GTTCTTGTCT CGCCTCAAGA ATCTTAAAGA AATGTACGTC GGCTACTTCA 700

***Clustal***  ********** ********** ********** ********** ********** 671

***BnA7.CLV1*** ACAGCTACAC CGGCGGCGTT CCGCCGGAGT TCGGTGAATT GACAAA**CCTA** 750

***BnC6.CLV1*** ACAGCTACAC CGGCGGCGTA CCGCCGGAGT TCGGTGAATT GACAAA**CCTA** 750

***Clustal***  ********** ********* ********** ********** ********** 720

***BnA7.CLV1*** **GAGGTCCTCG ACATGGCGA**G CTGTACTCTC ACAGGAGAGA TTCCGACAAC 800

**S6**

***BnC6.CLV1*** **GAGGTTCTCG ACATGGCGA**G CTGTACACTC ACGGGAGAGA TTCCGACGAC 800

***Clustal***  ***** **** ********** ****** *** ** ******* ******* ** 766

***BnA7.CLV1*** ACTAAGTAAT CTAAAACATT TGCACACTTT GTTTCTCCAC ATCAACAACT 850

***BnC6.CLV1*** TCTGAGTAAT CTAAAACATT TGCACACTTT GTTTCTCCAC ATCAACAACT 850

***Clustal***  ** ****** ********** ********** ********** ********** 814

***BnA7.CLV1*** TAACCGGAAA CATCCCACCA GAACTCTCCG GTTTAATCAG TTTAAAATCT 900

***BnC6.CLV1*** TAACCGGAAA CATCCCACCG GAACTCTCCG GTTTAATCAG CTTAAAATCT 900

***Clustal***  ********** ********* ********** ********** ********* 862

***BnA7.CLV1*** CTAGACCTCT CAATAAACCA GCTAACCGGA GAGATTCCTC AGAGCTTCAT 950

***BnC6.CLV1*** CTAGACCTCT CAATAAACCA GCTAACCGGA GAGATTCCTC AGAGCTTCAT 950

***Clustal***  ********** ********** ********** ********** ********** 912

***BnA7.CLV1*** CTCCCTGGGA AACATCACTC TCATCAACCT CTTCAGAAAC AATCTCCACG 1000

***BnC6.CLV1*** CTCCCTCTGG AACATCACTC TCATCAACCT CTTCAGAAAC AATCTCCACG 1000

***Clustal***  ****** * ********** ********** ********** ********** 959

***BnA7.CLV1*** GGCCGATACC TGAGTTCATC GGAGACATGC CGAACCTCCA AGTCCTCCAG 1050

***BnC6.CLV1*** GGCCSATACC TGAGTTCATC GGAGACATGC CGAACCTCCA AGTCCTCCAG 1050

***Clustal***  **** ***** ********** ********** ********** ********** 1008

***BnA7.CLV1*** GTGTGGGAGA ACAACTTCAC GCTAGAGCTA CCGGCGAATC TCGGCCGGAA 1100

***BnC6.CLV1*** GTGTGGGAGA ACAACTTCAC GCTAGAGCTA CCGGCGAATC TCGGCCGGAA 1100

***Clustal***  ********** ********** ********** ********** ********** 1058

***BnA7.CLV1*** CGGGAATCTG AAAAAGCTCG ACGTCTCTGA TAACCATCTC ACCGGACTCA 1150

***BnC6.CLV1*** CGGGAATCTG AAAAAGCTCG ACGTCTCTGA TAACCATCTC ACCGGACTCA 1150

***Clustal***  ********** ********** ********** ********** ********** 1108

***BnA7.CLV1*** TCCCCATGGA TTTGTGCAGA GGCGGGAAGC TGGAGACGCT CGTGCTCTCC 1200

***BnC6.CLV1*** TCCCCATGGA TTTGTGCAGA GGCGGGAAGC TGGAGACGCT SGTGCTCTCC 1200

***Clustal***  ********** ********** ********** ********** ********* 1157

***BnA7.CLV1*** AACAACTTCT TCTTCGGCTC GATCCCTGAG AAGCTAGGTC AATGCAAATC 1250

***BnC6.CLV1*** RACAACTTCT TCTTCGGCTC GATCCCTGAG AAGCTAGGTC GATGCAAATC 1250

***Clustal***  ********* ********** ********** ********** ********* 1205

***BnA7.CLV1*** GCTAAACAAG ATCAGAATCG TCAAGAATCT CCTCAACGGT ACGGTTCCGG 1300

***BnC6.CLV1*** GCTAAACAAG ATCAGAATCG TCAAGAATCT CCTCAACGGT ACGGTTCCGG 1300

***Clustal***  ********** ********** ********** ********** ********** 1255

***BnA7.CLV1*** AGGGATTATT CAATCTACCG CTCGTAACGA TCATCGAGCT CACCGATAAC 1350

***BnC6.CLV1*** CGGGACTATT CACTCTACCG CTCGTTACCA TCATCGAGCT CACCGATAAC 1350

***Clustal***  **** **** ** ******* ***** ** * ********** ********** 1300

***BnA7.CLV1*** TTCTTCTCCG GGGAGCTTCC GGGGGAGATG TCAGGCGACG TTCTCGATCA 1400

***BnC6.CLV1*** TTCTTCTCCG GGGAGCTTCC GGGGGAGATG TCAGGCGACC TTCTCGATCA 1400

***Clustal***  ********** ********** ********** ********* ********** 1349

***BnA7.CLV1*** TATCTACTTA TCTAACAATT GGTTTACCGG TTTAATCCCC CCGGCTATCG 1450

***BnC6.CLV1*** TATCTACTTA TCTAACAATT GGTTTACCGG TTTAATCCCC CCGGCTATCG 1450

***Clustal***  ********** ********** ********** ********** ********** 1399

***BnA7.CLV1*** GTAATTTTAA AAATCTACAA GATTTATTCT TAGACCGGAA CCGGTTTAGC 1500

***BnC6.CLV1*** GTAATTTTAA AAATCTACAG GATTTATTCT TAGACCGGAA CCGGTTTAGC 1500

***Clustal***  ********** ********* ********** ********** ********** 1448

***BnA7.CLV1*** GGGAATATCC CGAGGGAAGT TTTCGAGTTA AAGCATCTAA CGAAGATCAA 1550

***BnC6.CLV1*** GGGAATATTC CGAGAGAAGT TTTCGAGTTA AAGCATCTCA CTAAGATCAA 1550

***Clustal***  ******** * **** ***** ********** ******** * * ******** 1494

***BnA7.CLV1*** CACGAGTGCT AACAACCTAA CCGGCGACAT CCCTGACTCG ATCTCTCGCT 1600

***BnC6.CLV1*** CACGAGTGCT AACAACCTCA CCGGCGACAT CCCTGACTCG ATCTCKCGMT 1600

***Clustal***  ********** ******** * ********** ********** ***** ** * 1541

***BnA7.CLV1*** GCACTTCCTT AATCTCCGTC GATCTCAGCC GTAACCGAAT CGGCGGAGAT 1650

***BnC6.CLV1*** GCACTTCCTT AATCTCCGTC GATCTCAGCC GTAACCGAAT CGGCGGAGAT 1650

***Clustal***  ********** ********** ********** ********** ********** 1591

***BnA7.CLV1*** ATTCCTAAAG ACATCCACGA CGTGATTAAC TTAGGAACTC TAAATCTCTC 1700

***BnC6.CLV1*** ATCCCGAAAG ACATCCACGA CGTGATTAAC TTAGGAACTC TCAATCTCTC 1700

***Clustal***  ** ** **** ********** ********** ********** * ******** 1638

***BnA7.CLV1*** CGGGAATCAA CTCACCGGCT CGATCCCGAT CGGAATCGGG AAGATGACGA 1750

***BnC6.CLV1*** CGGGAATCAA CTCACCGGCT CGATCCCGAT CGGAATCGGG AAGATGACGA 1750

***Clustal***  ********** ********** ********** ********** ********** 1688

***BnA7.CLV1*** GCTTAACCAC TCTCGATCTC TCCTTCAACG ACCTCTCCGG GAGAGTCCCA 1800

***BnC6.CLV1*** GCTTAACCAC TCTCGATCTC TCCTTCAACG ACCTCTCGGG GCGAGTCCCA 1800

***Clustal***  ********** ********** ********** ******* ** * ******** 1736

***BnA7.CLV1*** CTCGGCGGCC AGTTCCTAGT CTTCAACGAC ACTTCCTTCG CCGGAAACCC 1850

***BnC6.CLV1*** CTCGGCGGCC AGTTCCTAGT CTTCAACGAC ACTTCCTTCG CCGGAAACCC 1850

***Clustal***  ********** ********** ********** ********** ********** 1786

***BnA7.CLV1*** TTACCTCTGC CTCCCTCACC ACGTCTCGTG CCTTACGCGT CCGGAACAAA 1900

***BnC6.CLV1*** TTACCTCTGC CTCCCTCGCC ACGTCTCGTG CCTCACGCGT CCCGGCCAAA 1900

***Clustal***  ********** ******* ** ********** *** ****** ** * **** 1831

***BnA7.CLV1*** CCTCCGATCG TATCCACACG GCTCTCTTCT CTCCGTCGAG GATCGTTATC 1950

***BnC6.CLV1*** CCTCCGATCG CATCCACACG GCGCTGTTCT CGCCGTCGAG GATCGCCATC 1950

***Clustal***  ********** ********* ** ** **** * ******** ***** *** 1875

***BnA7.CLV1*** ACGATCGTCG CAGCGATCAC GGCGTTGATC CTTATCAGCG TCGCGATTCG 2000

***BnC6.CLV1*** ACGATAATCG CAGCGGTCAC GGCGCTGATC CTCATCAGCG TCGCGATTCG 2000

***Clustal***  ***** *** ***** **** **** ***** ** ******* ********** 1920

***BnA7.CLV1*** TCAGATGAAC AAGAAGAAGC ACGAGAGATC TCTCTCCTGG AAGCTAACCG 2050

***BnC6.CLV1*** TCAGATGAAC AAGAAGAAGC ACGAGAGATC CCTCTCCTGG AAGCTAACCG 2050

***Clustal***  ********** ********** ********** ********* ********** 1969

***BnA7.CLV1*** CCTTCCAAAG ACTCGATTTC AAAGCGGAAG ACGTCCTCGA GTGTCTCCAG 2100

***BnC6.CLV1*** CCTTCCAGCG GCTCGATTTC AAGGCGGAAG ACGTCCTCGA GTGCCTCCAA 2100

***Clustal***  ******* * ********* ** ******* ********** *** ***** 2013

***BnA7.CLV1*** GAAGAGAACA TAATCGGCAA AGGCGGAGCG GGGATCGTCT ACCGCGGATC 2150

***BnC6.CLV1*** GAGGAGAACA TAATCGGCAA AGGCGGAGCG GGGATCGTCT ACCGCGGATC 2150

***Clustal***  ** ******* ********** ********** ********** ********** 2062

***BnA7.CLV1*** CATGCCGAAC AACGTCGACG TCGCGATCAA ACGCCTTGTA GGACGCGGAA 2200

***BnC6.CLV1*** CATGCCGAAC AACGTAGACG TCGCGATCAA ACGCCTCGTG GGACGCGGAA 2200

***Clustal***  ********** ***** **** ********** ****** ** ********** 2109

***BnA7.CLV1*** CAGGGAGGAG CGATCACGGA TTCACGGCGG AGATACAGAC GCTAGGGAGA 2250

***BnC6.CLV1*** CAGGGAGGAG CGATCACGGA TTCACGGCGG AGATTCAGAC GCTAGGGAGG 2250

***Clustal***  ********** ********** ********** **** ***** ********* 2157

***BnA7.CLV1*** ATCCGCCACC GTCATATAGT GAGACTCCTC GGATACGTGG CGAACAAGGA 2300

***BnC6.CLV1*** ATCCGCCACC GTCACATCGT GAGACTCCTC GGATACGTGG CGAACAAGGA 2300

***Clustal***  ********** **** ** ** ********** ********** ********** 2205

***BnA7.CLV1*** CACGAACCTG CTTCTCTACG AGTACATGCC TAACGGGAGC CTCGGGGAGC 2350

***BnC6.CLV1*** CACGAACCTG CTTCTCTACG AGTACATGCC TAACGGGAGC CTCGGCGAGC 2350

***Clustal***  ********** ********** ********** ********** ***** **** 2254

***BnA7.CLV1*** TTTTGCACGG ATCTAAAGGA GGTCATCTTC AGTGGGAGAC GAGGCACAGA 2400

***BnC6.CLV1*** TTTTGCACGG GTCTAAAGGA GGTCATCTTC AGTGGGAGAC GAGGCACAGA 2400

***Clustal***  ********** ********* ********** ********** ********** 2303

***BnA7.CLV1*** GTAGCCGTGG AAGCGGCGAA AGGACTGTGT TATCTTCATC ATGACTGTTC 2450

***BnC6.CLV1*** GTAGCCGTTG AAGCGGCGAA AGGACTGTGT TATCTTCACC ATGACTGTTC 2450

***Clustal***  ******** * ********** ********** ******** * ********** 2351

***BnA7.CLV1*** GCCGTTGATC TTGCATAGAG ACGTTAAGTC CAATAACATT TTACTGGACT 2500

***BnC6.CLV1*** GCCGTTGATC TTGCATAGAG ACGTTAAGTC CAATAACATT TTACTGGACT 2500

***Clustal***  ********** ********** ********** ********** ********** 2401

***BnA7.CLV1*** CTGATTTTGA GGCCCATGTT GCTGATTTTG GGCTTGCTAA GTTCTTAGTG 2550

***BnC6.CLV1*** CTGATTTTGA GGCCCATGTT GCTGATTTTG GGCTTGCTAA GTTCTTAGTG 2550

***Clustal***  ********** ********** ********** ********** ********** 2451

***BnA7.CLV1*** GACGGTGCTG CTTCTGAGTG TATGTCTTCG ATAGCTGGCT CCTATGGATA 2600

***BnC6.CLV1*** GACGGTGCTG CTTCCGAGTG TATGTCTTCG ATAGCTGGAT CCTATGGATA 2600

***Clustal***  ********** **** ***** ********** ******** * ********** 2499

***BnA7.CLV1*** CATCGCTCCA GGTTAGTTTT AAACATGTTT TAAATAACAA ATAATATGTA 2650

***BnC6.CLV1*** CATCGCTCCA GGTTAGTTT- AAACATGTTT TAAATAACAA ATAATATTTA 2649

***Clustal***  ********** ********* ********** ********** ******* ** 2547

***BnA7.CLV1*** TAAAACTAAC TATTGTTTGT TTTGGTTTTG AATTTTGATA GAGTATGCTT 2700

***BnC6.CLV1*** TAAAACTAAC TATTGTTTGT TTTGGTTTT- ------GATA GAGTATGCTT 2692

***Clustal***  ********** ********** ********* **** ********** 2590

***BnA7.CLV1*** ACACTCTCAA AGTGGATGAG AAGAGTGATG TGTATAGTTT CGGAGTGGTG 2750

***BnC6.CLV1*** ACACTCTCAA AGTGGATGAG AAGAGTGATG TTTATAGTTT TGGAGTGGTG 2742

***Clustal***  ********** ********** ********** * ******** ********* 2638

***BnA7.CLV1*** TTATTGGAGC TGATAGCTGG GAAGAAACCG GTTGGTGAGT TTGGGGAAGG 2800

***BnC6.CLV1*** TTATTGGAGC TGATAGCTGG GAAGAAACCG GTTGGTGAGT TTGGGGAAGG 2792

***Clustal***  ********** ********** ********** ********** ********** 2688

***BnA7.CLV1*** AGTGGATATA GTGAGGTGGG TGAGGAACAC GGAGGGTGAG ATACCTCAGC 2850

***BnC6.CLV1*** AGTGGATATA GTGAGGTGGG TGAGGAACAC GGAGGGTGAG ATACCTCAGC 2842

***Clustal***  ********** ********** ********** ********** ********** 2738

***BnA7.CLV1*** CGTCGGATGC AGCTACTGTT GTGGCGATCG TTGACCAGAG GTTGACTGGT 2900

***BnC6.CLV1*** CGTCGGATGC AGCTACTGTT GTTGCGATCG TCGACCAGAG GTTGACTGGT 2892

***Clustal***  ********** ********** ** ******* * ******** ********** 2786

***BnA7.CLV1*** TACCCGTTGA CTAGTGTGAT TCACGTGTTC AAGATAGCGA TGATGTGTGT 2950

***BnC6.CLV1*** TACCCGTTGA CTAGTGTGAT TCACGTGTTC AAGATAGCGA TGATGTGTGT 2942

***Clustal***  ********** ********** ********** ********** ********** 2836

***BnA7.CLV1*** GGAGGATGAG GCAGCGACAA GGCCGACGAT GAGGGAAGTT GTGCACATGC 3000

***BnC6.CLV1*** GGAGGATGAG GCAACGACAA GGCCGACGAT GAGGGAAGTT GTGCACATGC 2992

***Clustal***  ********** *** ****** ********** ********** ********** 2885

***BnA7.CLV1*** TCACTAACCC TCCCAAGTCC GTCACTAACT TGATCGCCTT CTGA 3044

***BnC6.CLV1*** TCACTAACCC TCCTAAGTCC GTGACTAACT TGATCGCCTT CTGA 3036

***Clustal***  ********** *** ****** ** ******* ********** **** 2927

(**b**)

***BnA7.CLV1*** MEMRLLKTHL LFLHLHYVIS ISLLCFSPCL ASTDMDHLLT LKSSMVGPNG 50

***BnC6.CLV1*** MEMRLLKTHL LFLHLHYVIS ILLLSFSPCF ASTDMDHLLT LKSSMVGPNG 50

***Clustal***  ********** ********** * **.****: ********** ********** 48

***BnA7.CLV1*** NGLHDWVHST SPAAHCSFSG VSCDGDARVI SLNVSFTPLF GTISPEIGML 100

***BnC6.CLV1*** HGLHDWVRSP SPSAHCSFSG VSCDGDARVI SLNVSFTPLF GTISPEIGML 100

***Clustal***  :******:*. **:******* ********** ********** ********** 97

***BnA7.CLV1*** NRLVNLTLAA NNFSGMLPLE MKSLTSLKVL NVSNNVNLNG TFPGEILTPM 150

***BnC6.CLV1*** DRLVNLTLAA NNFSGMLPLE MKSLTSLKVL NISNNVNLNG TFPGEILTPM 150

***Clustal***  :********* ********** ********** *:******** ********** 147

***BnA7.CLV1*** VDLEVLDAYN NNFTGPLPPE IPGLKKLRHL SLGGNFLTGE IPESYGDIQS 200

***BnC6.CLV1*** VDLEVLDAYN NNFTGPLPPE IPGLKKLRHL SLGGNFLTGE IPESYGDIQS 200

***Clustal***  ********** ********** ********** ********** ********** 197

***BnA7.CLV1*** LEYLGLNGAG LSGESPAFLS RLKNLKEMYV GYFNSYTGGV PPEFGELTNL 250

***BnC6.CLV1*** LEYLGLNGAG LSGESPAFLS RLKNLKEMYV GYFNSYTGGV PPEFGELTNL 250

***Clustal***  ********** ********** ********** ********** ********** 247

***BnA7.CLV1*** EVLDMASCTL TGEIPTTLSN LKHLHTLFLH INNLTGNIPP ELSGLISLKS 300

***BnC6.CLV1*** EVLDMASCTL TGEIPTTLSN LKHLHTLFLH INNLTGNIPP ELSGLISLKS 300

***Clustal***  ********** ********** ********** ********** ********** 297

***BnA7.CLV1*** LDLSINQLTG EIPQSFISLG NITLINLFRN NLHGPIPEFI GDMPNLQVLQ 350

***BnC6.CLV1*** LDLSINQLTG EIPQSFISLW NITLINLFRN NLHGPIPEFI GDMPNLQVLQ 350

***Clustal***  ********** ********* ********** ********** ********** 346

***BnA7.CLV1*** ---------- ---------- --LDVSDNHL TGLIPMDLCR GGKLETLVLS 378

***BnC6.CLV1*** VWENNFTLEL PANLGRNGNL KKLDVSDNHL TGLIPMDLCR GGKLETLVLS 400

***Clustal***  ******** ********** ********** 374

***BnA7.CLV1*** NNFFFGSIPE KLGQCKSLNK IRIVKNLLNG TVPEGLFNLP LVTIIELTDN 428

***BnC6.CLV1*** XNFFFGSIPE KLGRCKSLNK IRIVKNLLNG TVPAGLFTLP LVTIIELTDN 450

***Clustal***  ********* ***:****** ********** *** ***.** ********** 421

***BnA7.CLV1*** FFSGELPGEM SGDVLDHIYL SNNWFTGLIP PAIGNFKNLQ DLFLDRNRFS 478

***BnC6.CLV1*** FFSGELPGEM SGDLLDHIYL SNNWFTGLIP PAIGNFKNLQ DLFLDRNRFS 500

***Clustal***  ********** ***:****** ********** ********** ********** 471

***BnA7.CLV1*** GNIPREVFEL KHLTKINTSA NNLTGDIPDS ISRCTSLISV DLSRNRIGGD 528

***BnC6.CLV1*** GNIPREVFEL KHLTKINTSA NNLTGDIPDS ISRCTSLISV DLSRNRIGGD 550

***Clustal***  ********** ********** ********** ********** ********** 521

***BnA7.CLV1*** IPKDIHDVIN LGTLNLSGNQ LTGSIPIGIG KMTSLTTLDL SFNDLSGRVP 578

***BnC6.CLV1*** IPKDIHDVIN LGTLNLSGNQ LTGSIPIGIG KMTSLTTLDL SFNDLSGRVP 600

***Clustal***  ********** ********** ********** ********** ********** 571

***BnA7.CLV1*** LGGQFLVFND TSFAGNPYLC LPHHVSCLTR PEQTSDRIHT ALFSPSRIVI 628

***BnC6.CLV1*** LGGQFLVFND TSFAGNPYLC LPRHVSCLTR PGQTSDRIHT ALFSPSRIAI 650

***Clustal***  ********** ********** **:******* * ******** ********.* 619

***BnA7.CLV1*** TIVAAITALI LISVAIRQMN KKKHERSLSW KLTAFQRLDF KAEDVLECLQ 678

***BnC6.CLV1*** TIIAAVTALI LISVAIRQMN KKKHERSLSW KLTAFQRLDF KAEDVLECLQ 700

***Clustal***  **:**:**** ********** ********** ********** ********** 669

***BnA7.CLV1*** EENIIGKGGA GIVYRGSMPN NVDVAIKRLV GRGTGRSDHG FTAEIQTLGR 728

***BnC6.CLV1*** EENIIGKGGA GIVYRGSMPN NVDVAIKRLV GRGTGRSDHG FTAEIQTLGR 750

***Clustal***  ********** ********** ********** ********** ********** 719

***BnA7.CLV1*** IRHRHIVRLL GYVANKDTNL LLYEYMPNGS LGELLHGSKG GHLQWETRHR 778

***BnC6.CLV1*** IRHRHIVRLL GYVANKDTNL LLYEYMPNGS LGELLHGSKG GHLQWETRHR 800

***Clustal***  ********** ********** ********** ********** ********** 769

***BnA7.CLV1*** VAVEAAKGLC YLHHDCSPLI LHRDVKSNNI LLDSDFEAHV ADFGLAKFLV 828

***BnC6.CLV1*** VAVEAAKGLC YLHHDCSPLI LHRDVKSNNI LLDSDFEAHV ADFGLAKFLV 850

***Clustal***  ********** ********** ********** ********** ********** 819

***BnA7.CLV1*** DGAASECMSS IAGSYGYIAP ---------- ---------- EYAYTLKVDE 858

***BnC6.CLV1*** DGAASECMSS IAGSYGYIAP GFKHVLNNKY LNLLFVLVLI EYAYTLKVDE 900

***Clustal***  ********** ********** ********** 849

***BnA7.CLV1*** KSDVYSFGVV LLELIAGKKP VGEFGEGVDI VRWVRNTEGE IPQPSDAATV 908

***BnC6.CLV1*** KSDVYSFGVV LLELIAGKKP VGEFGEGVDI VRWVRNTEGE IPQPSDAATV 950

***Clustal***  ********** ********** ********** ********** ********** 899

***BnA7.CLV1*** VAIVDQRLTG YPLTSVIHVF KIAMMCVEDE AATRPTMREV VHMLTNPPKS 958

***BnC6.CLV1*** VAIVDQRLTG YPLTSVIHVF KIAMMCVEDE ATTRPTMREV VHMLTNPPKS 1000

***Clustal***  ********** ********** ********** *:******** ********** 949

***BnA7.CLV1*** VTNLIAF 965

***BnC6.CLV1*** VTNLIAF 1007

***Clustal***  ******* 956

**Fig. S3** Sequence alignment of the two *BnCLV1* gene copies. (**a**) Gene sequence alignment between the *BnA07.CLV1* and *BnC06.CLV1* copies. The target sequences (S3 to S6) are shown in bold and underlined with the PAM highlighted in red. (**b**) Protein sequence alignment between the *BnA07.CLV1* and *BnC06.CLV1* copies.

(**a**)

***BnA2.CLV2*** ATGGTGAAGA CTGCATATCT TACCCTCTTC TTCTT----- ---------- 35

***BnC2.CLV2*** ATGGTGAAGA CTGTATATTT TACTCTCTTC TTCTTTTTCT TCTTCTTCTT 50

***Clustal***  ********** *** **** * *** ****** ***** 32

***BnA2.CLV2*** ---CCTCTTC TCTCCTTCCT TGCTTCTAGC TCAATCACAG CCTCCAGAGA 82

***BnC2.CLV2*** CTTCCTCTTC TCTCCTTCGT TACTTCTAGC TCAATCACAG CCTCCAGAGA 100

***Clustal***  ******* ******** * * ******** ********** ********** 77

**S7**

***BnA2.CLV2*** TTGACCCACA AGACAAAG**CC TCGCTATTGA TACTCCGCGT T**TCGATTCAC 132

***BnC2.CLV2*** TTGACCCACT AGACAAAG**CC TCGCTATTGA TACTCCGCGT T**TCGATTCAC 150

***Clustal***  ********* ********** ********** ********** ********** 126

***BnA2.CLV2*** GACCCCAATC GAAGCTTATC GACATGGTAC GGCTCATCAT GTTCCAACTG 182

***BnC2.CLV2*** GACCCCAATC GAAGCTTATC GACATGGTAC GGCTCATCGT GTTCTAACTG 200

***Clustal***  ********** ********** ********** ******** * **** ***** 174

***BnA2.CLV2*** GACAGGTCTA GCTTGTCAGA ATCCGACCGG AAAAGTCGTC TCCCTCACTA 232

***BnC2.CLV2*** GACAGGTCTA GCTTGTCAGA ATCCGACCGG AAAAGTCATC TCCCTCACTA 250

***Clustal***  ********** ********** ********** ******* ** ********** 223

***BnA2.CLV2*** TACCCGCTTC GAATTTGTCC GGCCAGATTC ACCCGTCTCT CTGTAAGCTC 282

***BnC2.CLV2*** TACCCGCTGC GAATTTGTCC GGCCAGATTC ACCCGTCTCT CTGTAAGCTC 300

***Clustal***  ******** * ********** ********** ********** ********** 272

***BnA2.CLV2*** ACTTCACTTC AAATCTTGGA CCTTTCCGGA AACAATTTCT CCGGAAACAT 332

***BnC2.CLV2*** ACTTCACTTC AAATCTTGGA CCTTTCCGGA AACAATTTCT CCGGAAACAT 350

***Clustal***  ********** ********** ********** ********** ********** 322

***BnA2.CLV2*** CCCCGCTTGC TTCGGCGCGC TGCGTGATCT CAGAACACTC AATCTTAGCC 382

***BnC2.CLV2*** CCCCGCTTGC TTCGGCGCGC TGCGTTATCT CAGAACACTC AATCTTAGCC 400

***Clustal***  ********** ********** ***** **** ********** ********** 371

**S8**

***BnA2.CLV2*** GGAACGGTTT GGTTGGTTCG GTTCC**CGGAA CGTTCGTTAA CCTTAAGG**AG 432

***BnC2.CLV2*** GGAACAGTTT GGTTGGTTCG GTTCC**CGGAA CGTTCGTTAA CCTTAAGG**AG 450

***Clustal***  ***** **** ********** ********** ********** ********** 420

***BnA2.CLV2*** CTTAGAGAAT TAGTTCTAAG CGAGAATAGA GGTTTAAGAG GATCGGTTCC 482

***BnC2.CLV2*** CTTAGAGAAG TCGTTCTAAG CGAGAATAGA GGTTTAAGAG GATCGCTTCC 500

***Clustal***  ********* * ******** ********** ********** ***** **** 467

***BnA2.CLV2*** TCTCTGGTTT GGTGATTTGA TGTATCTCGA GAGGATTGAT CTTAGCTTCT 532

***BnC2.CLV2*** TCTCTTGGTC GGAGAATTGA TGAATCTCGA GAGAATTGAT CTAAGCTTCT 550

***Clustal***  ***** * * ** ** **** ** ******* *** ****** ** ******* 509

***BnA2.CLV2*** GTTTGTTTAG TGGAGAGATA CCTGAAACCT TGCTTTATTT AAAATCTCTA 582

***BnC2.CLV2*** GTTCCTTTCT TGGAGAGATA CCTGAAACCT TGCTTTATTT GAAATCTCTA 600

***Clustal***  *** *** ********** ********** ********** ********* 554

***BnA2.CLV2*** AAGGTTTTGA ATCTTGAGAG CAATAACATG ACCGGTACAC TTAGAGACTT 632

***BnC2.CLV2*** AAGTTTTTGA ATCTTGAGAG CAATAACATG ACCGGTACGT TTAGAGACTT 650

***Clustal***  *** ****** ********** ********** ******** ********** 601

***BnA2.CLV2*** CCAACAGCCG TTGGTTGTTC TCAACCTTGG TTCGAATCGG TTATCCGGTA 682

***BnC2.CLV2*** CCAACAGCCA TTGGTTGTTC TCAACCTTGC TTCGAATCGG TTTTCCGGTA 700

***Clustal***  ********* ********** ********* ********** ** ******* 648

***BnA2.CLV2*** CGCTGCCTTG TCTCTACGCC TCACGTCCAT CTCTAAGCGT TCTGAATCTC 732

***BnC2.CLV2*** CGCTGCCTTG TTTCTACGCC TCTCGTCCAT CTCTAAGCGT TCTGAATCTC 750

***Clustal***  ********** * ******** ** ******* ********** ********** 696

***BnA2.CLV2*** GCTGACAACT CATTAGTTGG TGGACTACCT TCTTGTTTGG GTTCTTTATC 782

***BnC2.CLV2*** GCTGACAACT CTCTGGTTGG TGGACTACCT TCTTGTTTGG GTTCTTTATC 800

***Clustal***  ********** * * ***** ********** ********** ********** 743

**S9**

***BnA2.CLV2*** AGAGCTGACT CATCTCAATC TCT**CCTTCAA TGCCTTCAGC TACGAG**ATAT 832

***BnC2.CLV2*** AGAGCTGACT CATCTCAATC TCT**CCTTCAA TGCCTTCAGC TACGAG**ATAT 850

***Clustal***  ********** ********** ********** ********** ********** 793

***BnA2.CLV2*** CTCCAAGGCT TATCTTCTCC GAGAAGCTCG TGATGCTTGA CTTGAGCCAC 882

***BnC2.CLV2*** CTCCAAGGCT TGTGTTCTCC GAGAAGCTCG TGATGCTTGA CTTGAGCCAC 900

***Clustal***  ********** * * ****** ********** ********** ********** 841

***BnA2.CLV2*** AACGGGTTCT CTGGTAGTCT CCCTAGCAGG ATCTCCGAAA CAACCGACAA 932

***BnC2.CLV2*** AACGGGTTCT CTGGTCGTCT CCCTAGCAGG ATCTCCGAAA CGACTGACAA 950

***Clustal***  ********** ***** **** ********** ********** * ** ***** 888

***BnA2.CLV2*** ACTCGGTCTG ATTCTTCTTG ATTTATCTTA CAACAGGTTC TCTGGTGGTA 982

***BnC2.CLV2*** ACTTGGTCTG ATTCTTCTTG ACCTCTCTTA CAATAGGTTC TCTGGTGGTA 1000

***Clustal***  *** ****** ********** * * ***** *** ****** ********** 933

***BnA2.CLV2*** TACCCTTGAG GATCACCGAG TTAACCAGCT TACAAGCATT GCGTCTCTCT 1032

***BnC2.CLV2*** TACCCTTGAG GATCACCGAG TTAACCAGAT TACAAGCATT GCGTCTCTCT 1050

***Clustal***  ********** ********** ******** * ********** ********** 982

***BnA2.CLV2*** CACAATCTTC TAACAGGAGA TATCCCTGCG CGAATAGGGA ACCTGACTTA 1082

***BnC2.CLV2*** CACAATCTTC TAACGGGAGA TATCCCTGCG AGAATCGGGA ACCTGACGTA 1100

***Clustal***  ********** **** ***** ********** **** **** ******* ** 1028

***BnA2.CLV2*** TCTCCAAGTC ATTGATCTTT CCCACAACGC GCTGACCGGA CCAATCCCTC 1132

***BnC2.CLV2*** TCTCCAAGTC ATTGATCTTT CCCACAACGC GCTGACCGGA CCAATCCCTC 1150

***Clustal***  ********** ********** ********** ********** ********** 1078

***BnA2.CLV2*** TCAACATCGT TGGCTGCTTT CAGTTGCTAG CTCTGATCAT CAGCAACAAC 1182

***BnC2.CLV2*** TCAACATCGT TGGCTGCTTT CAGTTGCTCG CTCTGATCAT CAGCAACAAC 1200

***Clustal***  ********** ********** ******** * ********** ********** 1127

***BnA2.CLV2*** AACCTCTCCG GCGAAATCCA GCCGGAGCTC GACGCGTTGG ACAGCCTGAA 1232

***BnC2.CLV2*** AACCTCTCCG GCGAAATCCA GCCGGAGCTC GACGCTTTGG ACAGCCTGAA 1250

***Clustal***  ********** ********** ********** ***** **** ********** 1176

**S10**

***BnA2.CLV2*** GATACTTGAC ATAAGCAGCA ACAGGATCTC TGGC**GAGATC CCGCTGACTC** 1282

***BnC2.CLV2*** GATACTTGAC ATAAGCAGCA ACAGGATCTC TGGC**GAGATC CCGCTGACTC** 1300

***Clustal***  ********** ********** ********** ********** ********** 1226

***BnA2.CLV2*** **TAGCCGG**CCT GAAGTCGCTA GAGATTGTGG ACATAAGCTC CAACAACCTC 1332

***BnC2.CLV2*** **TAGCCGG**TCT GAAGTCGCTA GAGATTGTGG ACATAAGCTC CAACAACCTC 1350

***Clustal***  ******* ** ********** ********** ********** ********** 1275

***BnA2.CLV2*** TCGGGAAGCC TTAACGAGGC CATTACCAAA TGGTCCAACC TCAAGTACCT 1382

***BnC2.CLV2*** TCGGGAAGCC TTAACGAGGC CATTACCAAG TGGTCCAACC TCAAGTACCT 1400

***Clustal***  ********** ********** ********* ********** ********** 1324

***BnA2.CLV2*** CTCTCTCGCA AGGAACAAGT TCAGCGGGAC GCTTCCTTCT TGGCTGTTCA 1432

***BnC2.CLV2*** CTCTCTCGCA AGGAACAAGT TCAGCGGAAC GCTTCCTTCT TGGCTGTTCA 1450

***Clustal***  ********** ********** ******* ** ********** ********** 1373

***BnA2.CLV2*** AGTTCGACAA GATCCAAATG ATGGACTACT CCAGCAACAG GTTCTCCTGG 1482

***BnC2.CLV2*** AGTTCGACAA GATCCAAATG ATGGACTACT CCAGCAACAG ATTCTCCTGG 1500

***Clustal***  ********** ********** ********** ********** ********* 1422

***BnA2.CLV2*** TTCATACCTG ACGATAACTT GAACAGCACA CGCTTCAAGG ATTTTCGGAC 1532

***BnC2.CLV2*** TTCATACCAG ACGATAACTT GAACAGCACC CGCTTCAAGG ATTTTCAGAC 1550

***Clustal***  ******** * ********** ********* ********** ****** *** 1469

***BnA2.CLV2*** CGCTGGATCT TCAGTGCCAC CGGGGAAAGT GGAGATCAAG ATATCAGCAA 1582

***BnC2.CLV2*** CGCTGGATCT TCAGTGCCAC CGGGGAAAGT AGAGATCAAG ATATCGGCAA 1600

***Clustal***  ********** ********** ********** ********* ***** **** 1517

***BnA2.CLV2*** ATGTGGTTGC TAAAGAGGAA CTAGCCTTCA GCTACGATCT CTTGTCAATG 1632

***BnC2.CLV2*** ATGTGGTTGC TAAAGACGAA CTAGCCTTCA GCTACGATCT CTTGTCGATG 1650

***Clustal***  ********** ****** *** ********** ********** ****** *** 1565

***BnA2.CLV2*** GTTGGGATCG ATCTTTCCGA CAATCTGTTA CACGGAGAAA TACCTGAAGC 1682

***BnC2.CLV2*** GTTGGGATCG ATCTTTCCGA CAATCTGTTA CACGGAGAAA TACCTGAAGC 1700

***Clustal***  ********** ********** ********** ********** ********** 1615

***BnA2.CLV2*** TTTGTTCAGA CAGAAGAACA TCGAGTACTT GAACTTGTCG TACAACTTCC 1732

***BnC2.CLV2*** TTTGTTCAGA CAGAAGAACA TCGAGTACTT GAACTTGTCG TACAACTTCC 1750

***Clustal***  ********** ********** ********** ********** ********** 1665

***BnA2.CLV2*** TTGAAGGCCA GCTTCCACGT TTAGAGAAGC TGCCGAGGCT AAAGGCCTTA 1782

***BnC2.CLV2*** TTGAGGGCCA GCTTCCACTT TTAGAGAAGC TGCCGAGGCT AAAGGCGTTA 1800

***Clustal***  **** ***** ******** * ********** ********** ****** *** 1712

***BnA2.CLV2*** GATCTTTCAC ACAACTCTCT CTCAGGTCAA GTCACTGGAA ATGTCTCAAC 1832

***BnC2.CLV2*** GATCTTTCAC ACAACTCTCT GTCAGGTCAA GTCACTGGAA ATGTCTCAAC 1850

***Clustal***  ********** ********** ********* ********** ********** 1761

***BnA2.CLV2*** TCCTCCAGGA CTGACTCTTC TGAATCTGTC TCACAACTGT TTCTCTGGAA 1882

***BnC2.CLV2*** TCCTCCAGGA CTGACTCTTC TGAATCTGTC TCACAACTGT CTCTCTGGAA 1900

***Clustal***  ********** ********** ********** ********** ********* 1810

***BnA2.CLV2*** TCGTTACTGA GAAAGAAGGG CTTGGAAAGT TTCCGGGGGC TTTGGTTGGG 1932

***BnC2.CLV2*** TCGTTACTGA GAAAGAAGGG CTTGGAAAGT TTCCAGGCGC TTTGGTTGGG 1950

***Clustal***  ********** ********** ********** **** ** ** ********** 1858

***BnA2.CLV2*** AACCCGGAAC TTTGTGTGGA AACTTCAGGG AACAAGTGTG ATCCTGCAGA 1982

***BnC2.CLV2*** AACCCGGAAC TTTGTGTGGA GTCTTCAGGA AACAAGTGTG ATCCTGCAAA 2000

***Clustal***  ********** ********** ******* ********** ******** * 1904

***BnA2.CLV2*** CATTGATGCA TCACAAGAGG AGATATATCA AAACGAGTTG GTGGAAGGAC 2032

***BnC2.CLV2*** CATTGATGCA TCAGAAGAGG AGATATATCA AAACGAGTTG GTGGAAGGAC 2050

***Clustal***  ********** *** ****** ********** ********** ********** 1953

***BnA2.CLV2*** CGATATCGAT TTGGATATTC TGCTTAAGCG CCTTTATTAG CTTTGATTTT 2082

***BnC2.CLV2*** CGATATCGAT TTGGATATTC TGCTTAAGCG CGTTTATTAG CTTTGATTTT 2100

***Clustal***  ********** ********** ********** * ******** ********** 2002

***BnA2.CLV2*** GGAGTGTTGG GTATCTTCTG CTCATCTCGT GCTCGGAGTT ATATTCTCCA 2132

***BnC2.CLV2*** GGGGTGTTGG GTATCTTCTG CTCATCTCGT GCTCGGAGTT ATGTTCTCCA 2150

***Clustal***  ** ******* ********** ********** ********** ** ******* 2050

***BnA2.CLV2*** GACCAAAGGT TAA 2145

***BnC2.CLV2*** GACCAAAGGT TAA 2163

***Clustal***  ********** *** 2063

(**b**)

***BnA2.CLV2*** MVKTAYLTLF FF------LF SPSLLLAQSQ PPEIDPQDKA SLLILRVSIH 44

***BnC2.CLV2*** MVKTVYFTLF FFFFFFFFLF SPSLLLAQSQ PPEIDPLDKA SLLILRVSIH 50

***Clustal***  ****.*:*** ** ** ********** ****** *** ********** 42

***BnA2.CLV2*** DPNRSLSTWY GSSCSNWTGL ACQNPTGKVV SLTIPASNLS GQIHPSLCKL 94

***BnC2.CLV2*** DPNRSLSTWY GSSCSNWTGL ACQNPTGKVI SLTIPAANLS GQIHPSLCKL 100

***Clustal***  ********** ********** *********: ******:*** ********** 92

***BnA2.CLV2*** TSLQILDLSG NNFSGNIPAC FGALRDLRTL NLSRNGLVGS VPGTFVNLKE 144

***BnC2.CLV2*** TSLQILDLSG NNFSGNIPAC FGALRYLRTL NLSRNSLVGS VPGTFVNLKE 150

***Clustal***  ********** ********** ***** **** *****.**** ********** 140

***BnA2.CLV2*** LRELVLSENR GLRGSVPLWF GDLMYLERID LSFCLFSGEI PETLLYLKSL 194

***BnC2.CLV2*** LREVVLSENR GLRGSLPLLV GELMNLERID LSFCSFLGEI PETLLYLKSL 200

***Clustal***  ***:****** *****:** . *:** ***** **** * *** ********** 185

***BnA2.CLV2*** KVLNLESNNM TGTLRDFQQP LVVLNLGSNR LSGTLPCLYA SRPSLSVLNL 244

***BnC2.CLV2*** KFLNLESNNM TGTFRDFQQP LVVLNLASNR FSGTLPCFYA SRPSLSVLNL 250

***Clustal***  *.******** ***:****** ******.*** :******:** ********** 233

***BnA2.CLV2*** ADNSLVGGLP SCLGSLSELT HLNLSFNAFS YEISPRLIFS EKLVMLDLSH 294

***BnC2.CLV2*** ADNSLVGGLP SCLGSLSELT HLNLSFNAFS YEISPRLVFS EKLVMLDLSH 300

***Clustal***  ********** ********** ********** *******:** ********** 283

***BnA2.CLV2*** NGFSGSLPSR ISETTDKLGL ILLDLSYNRF SGGIPLRITE LTSLQALRLS 344

***BnC2.CLV2*** NGFSGRLPSR ISETTDKLGL ILLDLSYNRF SGGIPLRITE LTRLQALRLS 350

***Clustal***  ***** **** ********** ********** ********** ** ******* 331

***BnA2.CLV2*** HNLLTGDIPA RIGNLTYLQV IDLSHNALTG PIPLNIVGCF QLLALIISNN 394

***BnC2.CLV2*** HNLLTGDIPA RIGNLTYLQV IDLSHNALTG PIPLNIVGCF QLLALIISNN 400

***Clustal***  ********** ********** ********** ********** ********** 381

***BnA2.CLV2*** NLSGEIQPEL DALDSLKILD ISSNRISGEI PLTLAGLKSL EIVDISSNNL 444

***BnC2.CLV2*** NLSGEIQPEL DALDSLKILD ISSNRISGEI PLTLAGLKSL EIVDISSNNL 450

***Clustal***  ********** ********** ********** ********** ********** 431

***BnA2.CLV2*** SGSLNEAITK WSNLKYLSLA RNKFSGTLPS WLFKFDKIQM MDYSSNRFSW 494

***BnC2.CLV2*** SGSLNEAITK WSNLKYLSLA RNKFSGTLPS WLFKFDKIQM MDYSSNRFSW 500

***Clustal***  ********** ********** ********** ********** ********** 481

***BnA2.CLV2*** FIPDDNLNST RFKDFRTAGS SVPPGKVEIK ISANVVAKEE LAFSYDLLSM 544

***BnC2.CLV2*** FIPDDNLNST RFKDFQTAGS SVPPGKVEIK ISANVVAKDE LAFSYDLLSM 550

***Clustal***  ********** *****:**** ********** ********:* ********** 531

***BnA2.CLV2*** VGIDLSDNLL HGEIPEALFR QKNIEYLNLS YNFLEGQLPR LEKLPRLKAL 594

***BnC2.CLV2*** VGIDLSDNLL HGEIPEALFR QKNIEYLNLS YNFLEGQLPL LEKLPRLKAL 600

***Clustal***  ********** ********** ********** ********* ********** 580

***BnA2.CLV2*** DLSHNSLSGQ VTGNVSTPPG LTLLNLSHNC FSGIVTEKEG LGKFPGALVG 644

***BnC2.CLV2*** DLSHNSLSGQ VTGNVSTPPG LTLLNLSHNC LSGIVTEKEG LGKFPGALVG 650

***Clustal***  ********** ********** ********** :********* ********** 630

***BnA2.CLV2*** NPELCVETSG NKCDPADIDA SQEEIYQNEL VEGPISIWIF CLSAFISFDF 694

***BnC2.CLV2*** NPELCVESSG NKCDPANIDA SEEEIYQNEL VEGPISIWIF CLSAFISFDF 700

***Clustal***  *******:** ******:*** *:******** ********** ********** 680

***BnA2.CLV2*** GVLGIFCSSR ARSYILQTKG 714

***BnC2.CLV2*** GVLGIFCSSR ARSYVLQTKG 720

***Clustal***  ********** ****:***** 700

**Fig. S4** Sequence alignment of the two *BnCLV2* gene copies. (**a**) Gene sequence alignment between the *BnA02.CLV2* and *BnC02.CLV2* copies. The target sequences (S7 to S10) are shown in bold and underlined with the PAM highlighted in red. (**b**) Protein sequence alignment between the *BnA02.CLV2* and *BnC02.CLV2* copies.


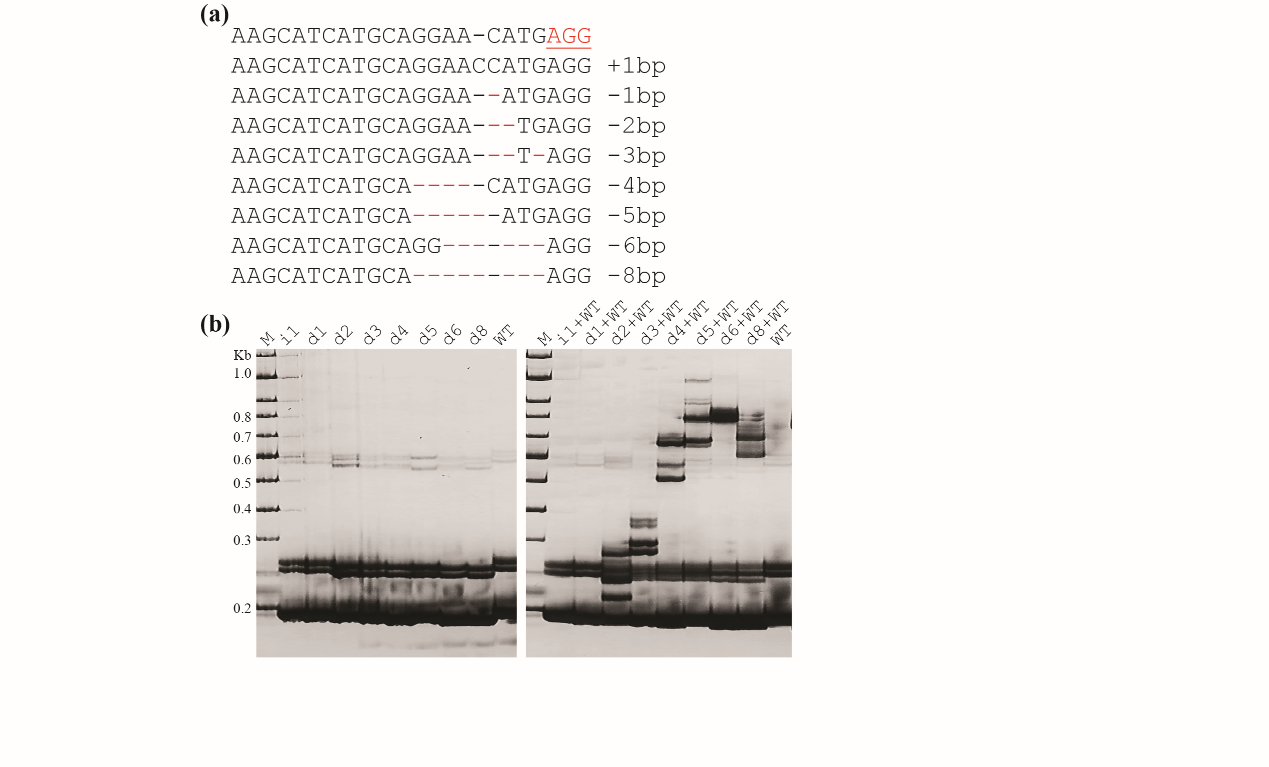


**Fig. S5** Detection of a series of mutations with different indel sizes using a PAGE-based method. (**a**) The S1 target sequences of a set of indel plasmids with different indel sizes used for the PAGE-based assay. (**b**) These indels were PCR ampliﬁed and detected using a PAGE analysis. PCR products with homozygous indels are shown on the left, and PCR products with heterozygous indels are shown on the right. d#, # of base pairs (bp) deleted from the target site; i#, # of bp inserted at the target site. The length of the PCR products was approximately 186 bp.


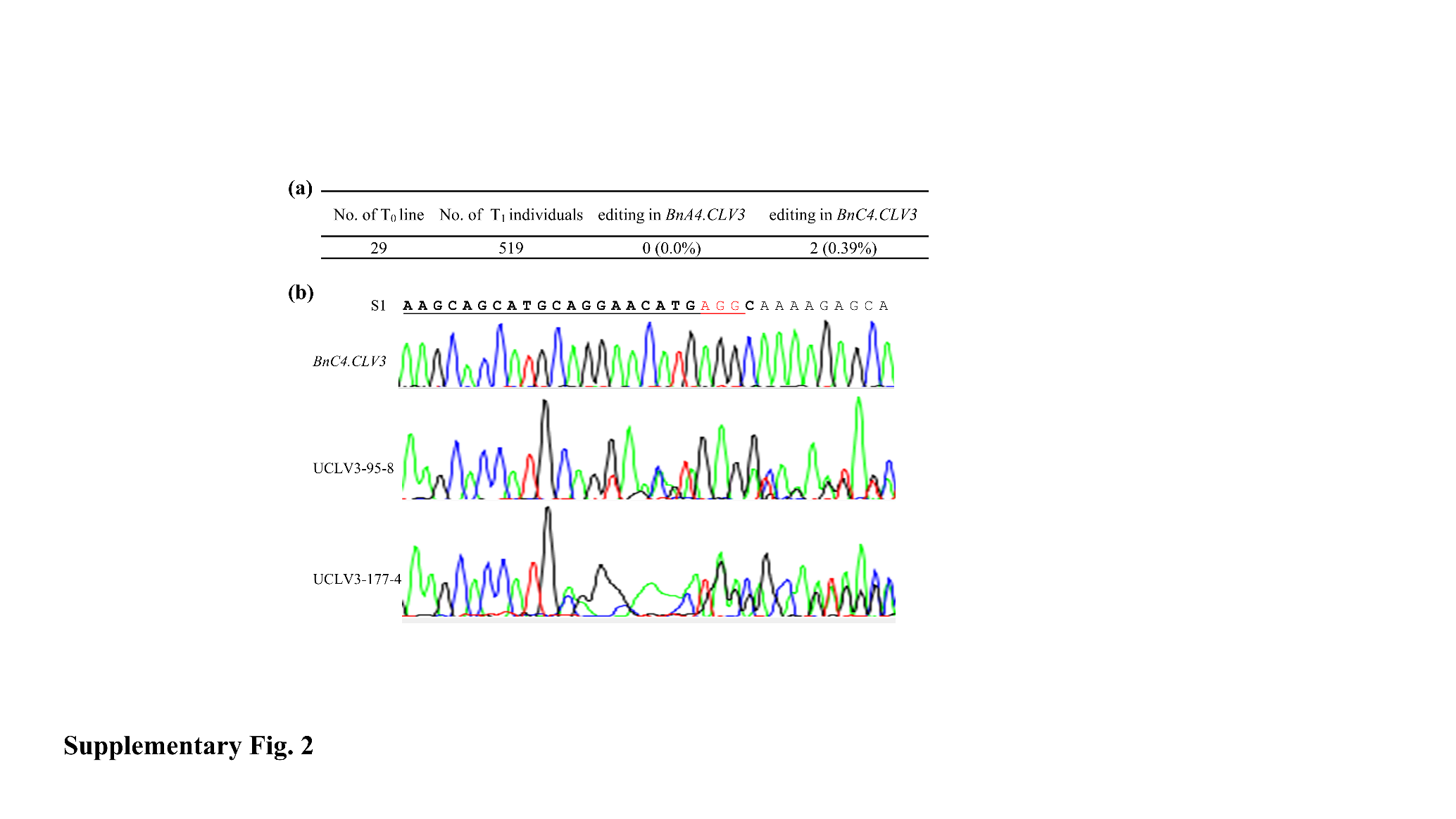


**Fig. S6** Novel mutations were detected in the T_1_ progeny with T-DNA transmission. (**a**) Mutant detection at the S1 site using a PAGE-based method in 519 T_1_ plants from 29 non-edited T_0_ lines. (**b**) Sequencing chromatogram of the S1 target site in two T_1_ lines to detect new mutations. Heterozygous mutants were determined according to the presence of double peaks in the sequencing chromatogram beginning at the site of the indel.


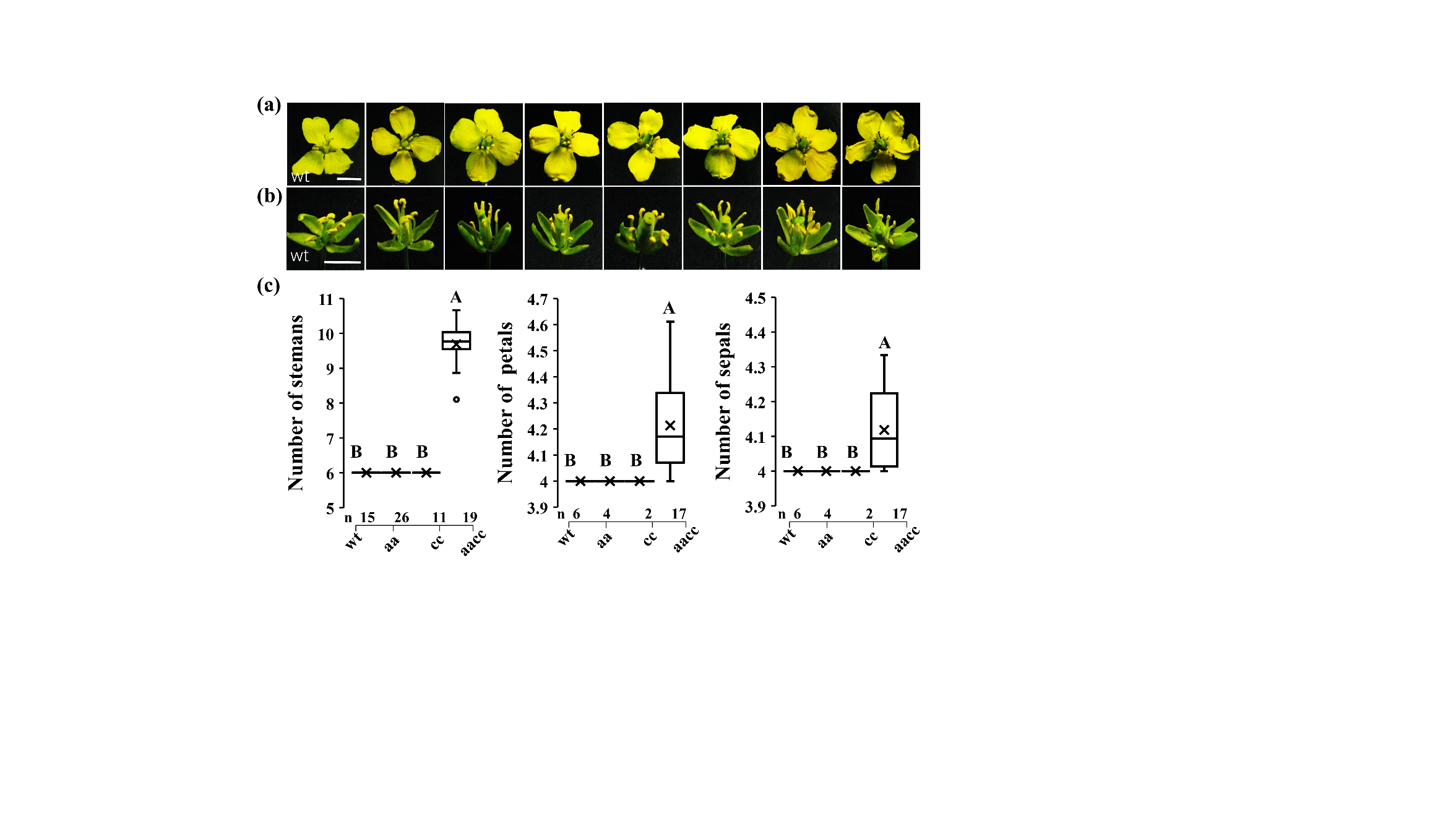


**Fig. S7** Variations in the floral organs in the double homozygous mutants of *BnCLV3.* (**a, b**) Flowers in the control WT had 6 stamens, 4 petals and 4 sepals; flowers in double mutants had increased numbers of sepals, petals and stamens. (**c**) Statistical analysis of the numbers of different floral organs in the WT and single and double mutants. The *middle line,* the median; the *plus sign,* the mean; the *box,* the range from the 25th to 75th percentiles of the total data; and the *whiskers,* the interquartile range. The number of measured individuals is indicated at the bottom (more than 30 flowers for each plant). Uppercase letters indicate a significant difference at the 0.01 probability level. WT, wild-type; aa, homozygous mutation in *BnA04.CLV3*; cc, homozygous mutation in *BnC04.CLV3*; aacc, homozygous mutation both in *BnA04.CLV3* and *BnC04.CLV3*.

**
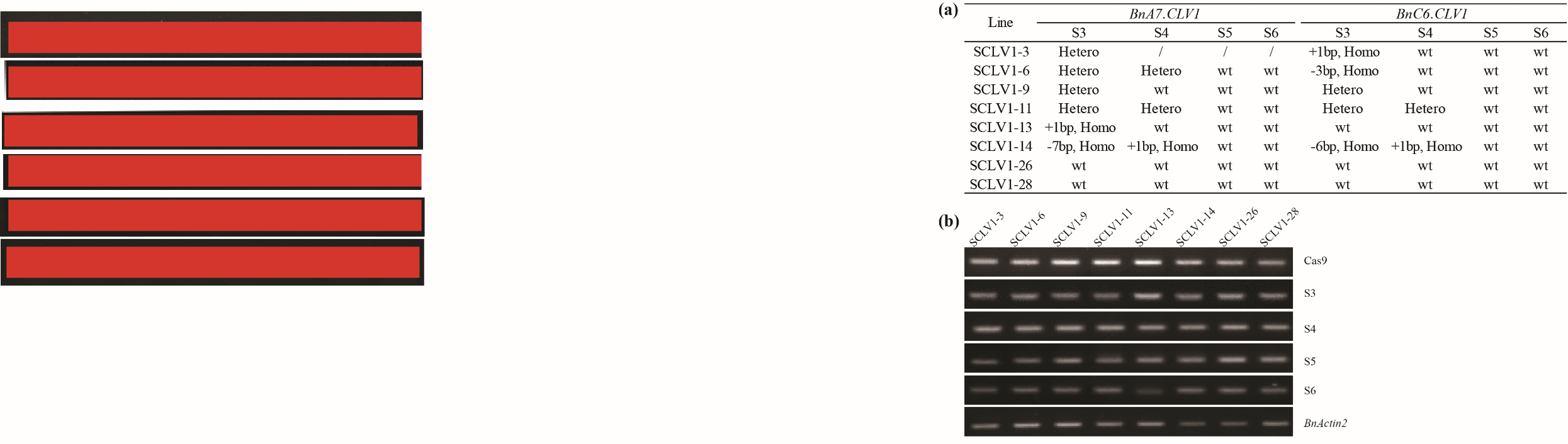
**

**Fig. S8** Expression of *Cas9p* and sgRNAs in SCLV1. (**a**) The genotypes of eight T_0_ plants used for the gene expression analysis. Hetero, heterozygous mutation; Homo, homozygous mutation; WT, wild-type; “/”, unknown. (**b**) Expression of *Cas9p* and sgRNAs detected using RT-PCR. *BnActin2* was used as the internal control. "S#" represents the specific targets of *BnCLVs* (details are provided in Fig. 1).
